# Dynamic expression of miRNAs across immature and adult stages of the malaria mosquito *Anopheles stephensi*

**DOI:** 10.1186/s13071-015-0772-y

**Published:** 2015-03-25

**Authors:** Shanu Jain, Vandita Rana, Adak Tridibes, Sujatha Sunil, Raj K Bhatnagar

**Affiliations:** Insect Resistance Group, International Centre for Genetic Engineering and Biotechnology, New Delhi, India; National Institute of Malaria Research, Dwarka, New Delhi India

**Keywords:** Mosquito development, microRNA, NGS, Antagomir, Degradome sequencing

## Abstract

**Background:**

MicroRNAs are small non-coding RNAs that are involved in various biological processes including insect development. *Anopheles stephensi* serves as primary vector of malaria parasite in Asia and exhibits holometabolous life cycle that involves four different stages of development. Regulation and role of mosquito miRNAs during various stages of mosquito development remain largely unknown.

**Methods:**

High throughput small RNA sequencing was employed for identification and profiling of miRNAs across immature and adult stages of malaria vector*,* which were further validated using Northern hybridization and real time PCR. Target prediction and pathway analysis was carried out to understand the role of regulated miRNAs in insect development. Degradome sequencing was employed to identify cleaved targets of some regulated miRNAs. Loss of function strategy was employed for miR-989 to understand its probable role in female reproductive process.

**Results:**

Small RNA sequencing and data analysis revealed 111 and 14 known and novel miRNAs respectively across all stages of *Anopheles stephensi.* Nine miRNAs showed gender specific regulation across different stages of mosquito development. Analysis of miRNAs revealed regulation of 24 and 26 miRNAs across different stages of male and female mosquito development respectively. mRNA targets and significant pathways targeted by regulated miRNAs were identified for each stage of mosquito development. Degradome sequencing revealed twenty nine cleaved targets of insect miRNAs. MicroRNA-989 showed significant up-regulation in the adult female as compared to adult male mosquito. Knockdown of miR-989 expression in adult female using miRNA specific antagomir affected targets playing roles in protein binding, proteolysis and nucleic acid binding in ovary tissue of female mosquito post blood feeding.

**Conclusions:**

This is the first comprehensive effort to understand regulation of *Anopheles stephensi* miRNAs across developmental stages of male and female mosquito. Preliminary role of regulated miRNAs in mosquito development was revealed by target prediction and pathway analysis. MicroRNA-989 emerged to have important roles in adult female mosquitoes showing significant up-regulation which was further studied using miR-989 specific antagomir. This study provides insights into mosquito development and reproductive process and has implications for effective control of mosquito population required for reducing spread of mosquito-borne infectious diseases.

**Electronic supplementary material:**

The online version of this article (doi:10.1186/s13071-015-0772-y) contains supplementary material, which is available to authorized users.

## Background

Mosquitoes exhibit holometabolous life cycle that proceeds through four different life stages. Eggs laid by blood fed female mosquitoes hatch into larvae that metamorphosis into pupae and finally to imago or adult mosquitoes. These stages exhibit distinctive morphological and physiological differences as depicted by their choice of different ecological niches. After hatching, larva grows and periodically sheds its old cuticle by the process of ecdysis. This process results in larval growth through successive instar to reach fourth instar stage of development. Larva metamorphosis into pupa involves almost complete histolysis and phagocytosis of the larval tissues. Pupa is a non feeding inactive stage during which reconstruction of histolysed tissue results in formation of organs of an adult mosquito.

Such complete metamorphosis involves complex interplay of ecdysteroid and juvenile hormone activities and regulation of transcriptional events [1,2].

MicroRNAs are non protein coding small RNAs of length 18–24 nucleotides that are produced by plants, animals and viruses [3-5]. They regulate numerous biological processes such as growth and development, differentiation, disease progression, apoptosis and immunity [6]. Such regulation is mediated by miRNA binding to 3’UTR sequence of target gene and regulating their expression either by target cleavage or translation repression [7]. Repertoire of miRNAs in different mosquito species has been reported [8-10]. Few of them have been functionally characterized and were shown to play critical role in insect reproduction and immunity against pathogens [11-13]. Few studies have focused on understanding the regulation of miRNAs across developmental stages of mosquito [14,15]. Previous studies have identified miRNAs in *An.stephensi* and studied miRNA regulation in blood fed and post *Plasmodium* infected female mosquito [9,16]. Understanding the regulation of miRNAs across immature stages of *An.stephensi,* an important disease vector in Asia remains largely unfulfilled.

In this study, we employed next generation small RNA sequencing to identify miRNAs that are regulated across *Anopheles stephensi* immature stages. We studied miRNA differences between male and female mosquito during larval, pupal and adult stages of their development, as differences between the genders render the female mosquito fit to serve as vector for parasite transmission. MicroRNAs differentially expressed during metamorphosis from larval to pupal and then from pupal to adult stages were identified. Characterization of these miRNAs may offer insight into vital processes such as ecdysis, histolysis and generation of adult organs during mosquito life cycle. Additionally, a number of novel mosquito-specific miRNAs were also discovered. Further, mRNA targets were predicted to understand their role in mosquito development. Antagomir injections and degradome sequencing were employed for the first time in insects to identify mRNA targets cleaved by regulated miRNAs in ovary tissue of female mosquito. Ovary specific cleavage of targets highlights towards their role in insect reproduction. Understanding the functions of these regulated miRNAs will provide useful insights in mosquito biology and is capable of deciphering ways to control mosquito-borne infectious diseases.

## Methods

### Ethics statement

Animal experiments were performed in accordance with National animal ethics guidelines of the Government of India after approval by Institutional Animal Ethics Committees of International Centre for Genetic Engineering & Biotechnology, New Delhi (Permit number: ICGEB/AH/2011/01/IR-8).

### Rearing of mosquito

All developmental stages of *Anopheles stephensi* including egg, larva, pupa and adult mosquitoes were reared under optimum temperature at 28 ± 2°C and 70 -75% humidity in an insectary. Laid eggs were transferred to the enamel trays and were allowed to hatch into a first instar larvae. Larvae were fed on fish food and were allowed to grow from first instar to fourth instar larvae. Fourth instar larvae transforms into pupae, which were collected and kept into cloth cages. Emerging adult mosquitoes were maintained in same cages and were fed on water soaked raisins and 1% glucose soaked cotton pads. To obtain next generation of eggs, 5–6 days old female mosquitoes were fed for two hours on mice as a source of blood meal. Female mosquitoes developed and laid eggs three days post blood feeding in a bowl of sterilized water.

### Sample collection and RNA isolation

Mosquitoes at different stages of their development, namely fourth instar larvae, pupae and 5–6 days old adult mosquitoes, with a minimum sample size of 100 numbers in each group for both genders were collected. The samples were collected a minimum of three times during different rearing cycles for each stage of development. Total RNA enriched in small RNA population was extracted using miRNeasy kit (Qiagen) as per the manual’s protocol. Total RNA from all biological replicates were pooled during small RNA library preparation. Collecting samples from different cycles of mosquito rearing would help in nullifying changes induced by possibly altered rearing conditions and hence served as biological replicates. Quality and quantity of RNA was checked by using Agilent 2100 Bioanalyzer RNA Nano 6000 kit.

### Small RNA sequencing library preparation, sequencing and data analysis

Small RNA libraries were prepared from total RNA extracted from all six samples viz., male and female fourth instar larva, male and female pupa and 5–6 days old male and female adult mosquitoes. Small RNA library preparation and post sequencing data analysis was carried out as described in [9]. Briefly, Illumina Trueseq libraries were prepared by ligating adaptors to both side of the RNA sequence followed by reverse transcription. Adaptors ligated fragments were PCR amplified and were 14–160 bps fragments were purified using 6% TBE PAGE gel. Prepared libraries were then sequenced using Illumina Genome Analyzer II.

In-house developed data analysis pipeline was used for analysing post sequencing raw data as described in [8]. Briefly, raw sequences were filtered and sequences with length >18 nt were selected. Total number of unique reads were identified in each library, which were then aligned against mature miRNA sequence downloaded from mirbase database using bowtie with zero mismatch as a parameter [17,18]. Unmatched sequences were used for novel miRNAs identification. Unmatched sequences were mapped on to pre-miRNA, ncRNA database [19] and coding region of genes of different mosquito species downloaded from vectorbase [20]. Unmatched sequences were then matched to *An.stephensi* genome. Matched sequences as well as 75 nt sequences flanking on either side of the mapped sequences were extracted. These sequences were folded into secondary structures using RNAfold [21] and their folding energies were calculated using RNAplot [21]. Sequences folding into hairpin loop structure, folding energy < −20 kcal/mol and small sequence mapping on the arm of secondary structure were classified as novel miRNAs [8].

### Differential expression of miRNAs and statistical analysis

Total number of reads for each miRNA in all the six libraries were obtained by using in house Perl script. Reads were normalised by calculating tags per million of total RNA reads (TPM) for each miRNA in all the six libraries. TPM data obtained was then fed into edgeR module for identification of differentially expressed genes between different developmental stages. The P value cut off was performed on the data with the significance threshold selected as 0.05. The final set of miRNAs that were considered to be significantly regulated were selected on the basis of three criteria, namely, > two fold change in expression between compared stages, P value ≤ 0.05, and TPM >10.

### Antagomir injections in mosquitoes

Antagomir for miR-989 was synthesized complementary to mature miRNA sequence with 2’-O-methyl (2’-OMe) group at each base and also with 3’ cholesterol group (Dharmacon, USA). Scrambled RNA was synthesized with same modifications and was used as a negative control. 4–5 days old female mosquitoes were divided into three batches of 200 mosquitoes each. First batch was injected with 69 nl of PBS. Second and third batch of mosquitoes were injected with 69 nl of 100 uM scrambled RNA and miR-989 antagomir each. Mosquitoes were allowed to recover for two days and were fed on uninfected mice blood. Ovaries were dissected out of female mosquitoes 24 hours post blood feeding and were then stored in Trizol at −80°C till RNA extraction. Knockdown of miRNA expression post injection was checked by miRNA qRT-PCR.

### Quantitative RT-PCR

Expression profiling of miRNAs across three immature stages in both genders was carried out by quantitative RT-PCR. Ten ng of RNA was reverse transcribed using cDNA synthesis kit (Exiqon). Real time reactions were set up using 1:80 diluted cDNA, custom miRNA LNA PCR primer sets (Exiqon) and SYBR green master mix (Exiqon) following manufacturer’s instructions in ABI one step detection system. Experiments were conducted a minimum of two times, with each experiment set up in triplicates for all developmental stages. 5.8 s rRNA was used as an endogenous control for miRNA expression profiling. Expression levels were then calculated against adult female mosquito as a calibrator using 2^-ΔΔC^_T_ method.

### Northern hybridization

Digoxigenin-labelled antisense miRCURY LNA probes (Exiqon) were used for detection of miRNAs by Northern hybridization. 10ug of total RNA isolated from 5–6 days old female mosquito was run using 15% denaturing polyacrylamide gels (PAGE). After the run was completed, gels were stained with ethidium bromide and RNA quality was checked using transilluminator. RNA was then transferred to the nylon membrane using semi-dry transfer apparatus. Nylon membrane with the transferred RNA was subjected to EDC crosslinking. Crosslinked membranes were pre-hybridized in a rotating hybridization oven for 30 min at 37 deg in hybridization buffer. Overnight hybridization was done in same buffer at 37 deg with the final concentration of 0.5 nM miRCURY LNA probe (Exiqon). The membranes were washed twice for 10 min each in a low stringent buffer (2X SSC, 0. 1% SDS) at RT and then once in washing buffer (1X SSC) at RT. The membranes were incubated for 3 h in blocking buffer (Roche) followed by 30 min incubation with Anti-DIG-alkaline phosphatase fragment antibody (Roche) in blocking buffer. The membranes were then washed in DIG washing buffer at RT for 15 min each and then incubated for 5 min in development buffer. CSPD substrate (1:100 diluted in development buffer) was applied on to the membranes and incubated in dark for 10 min. Chemiluminescence signal was then measured in a Fluorchem machine (Protein Simple) to detect miRNA on the membrane.

### MicroRNA target prediction and identification of enriched pathways

mRNA targets of miRNAs regulated across developmental stages were predicted using RNAhybrid software [22]. 3’UTR sequence of *An.stephensi* genes were downloaded from vector base. Mature miRNA sequence (fasta) and downloaded 3’UTR sequence were used as input data files in RNAhybrid. Targets were predicted using following parameters (i) Perfect miRNA seed complementarity with 3’UTR sequence (ii) P value < 0.05 (iii) miRNA:mRNA binding energy < −20 kcal/mol. Targets predicted using these criteria’s were selected for pathway analysis. Orthologs of these targets present in *An.gambiae* were fetched from vector base. Targets ortholog of each miRNA cluster were analysed by NIH DAVID resource [23]. mRNA targets of miRNA cluster were analysed using functional annotation clustering with EASE score of 0.1. Enrichment score for each cluster was calculated to highlight enriched role of group members in the given study.

### Degradome sequencing for miRNA target identification

Total RNA was extracted from ovaries of mosquitoes injected with PBS, scrambled RNA and miR-989 antagomir as described before. Degradome library construction and sequencing was performed by LC Sciences (Houston, USA). Data analysis was performed in the lab using an in-house developed pipeline. Raw reads obtained after sequencing were processed to remove low quality reads. Adaptor sequences were then trimmed from the raw reads. Resulting 20–25 nt sequences were selected for further analysis. Potentially cleaved miRNA target were identified using Parallel Analysis of RNA Ends (PAREsnip) [24]. Degradome sequences, cDNA of *An. stephensi* downloaded from Vectorbase and mature miRNA sequences were provided as an input to the PAREsnip software. Target plots (T-plots) were generated showing relative abundance of fragments mapping at the miRNA target site relative to the abundance of fragments found at other sites on the transcript. Depending on this, identified targets were grouped into five categories. In category 0, maximum number of degraded sequences (>1) were present at one site on the transcript. In category 1, two or more sites were present on the transcripts where degraded sequences (>1) map with the same abundance. If abundance at a site was less than maximum but greater than median abundance for the transcript, target was classified as category 2. In category 3, abundance at a position was less or equal to the median value for that transcript. Category 4 was classified with only one raw read at the position.

## Results

MicroRNA dynamics was studied during metamorphosis of both male and female *An.stephensi* mosquito. Six small RNA libraries enriched in miRNAs were constructed from larva male (LM), larva female (LF), pupa male (PM), pupa female (PF) adult male (AM) and adult female (AF) mosquitoes. Constructed libraries were sequenced using sequencing by synthesis technology, (Illumina Inc.). Raw data generated for all six libraries was analysed using in-house data analysis pipeline [8].

### Deep sequencing of small RNA libraries

**Figure 1:**
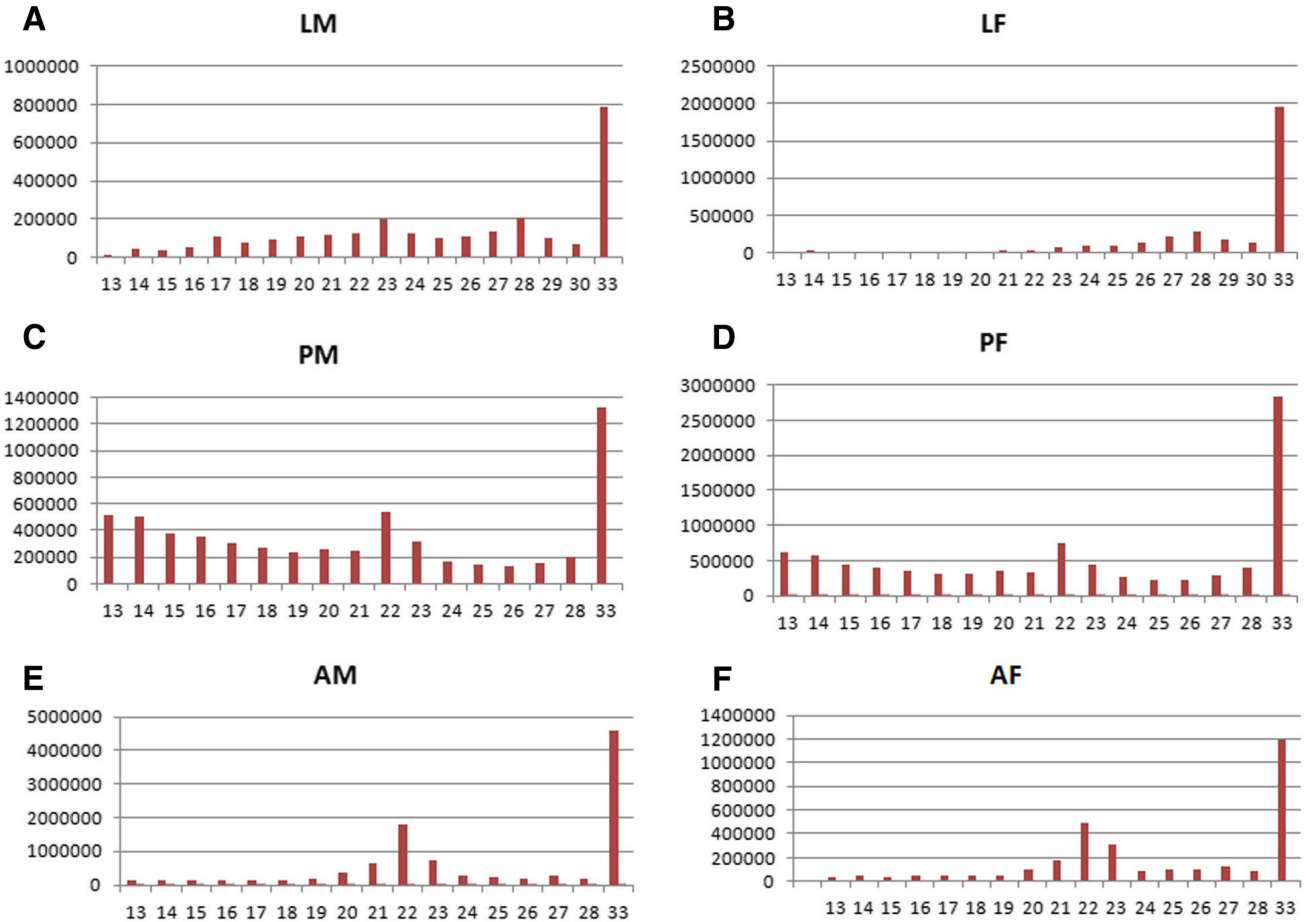
**Size distribution of small RNA reads in deep sequenced libraries.** Column graphs showing different sizes of small RNA reads in **(A)** larva male (LM), **(B)** larva female (LF), **(C)** pupa male (PM), **(D)** pupa female (PF), **(E)** adult male (AM) and **(F)** adult female (AF) mosquito.

Sequencing by synthesis of six different libraries yielded 7.2 × 10^7^ raw reads. After adaptor trimming, total of 5.9 × 10^7^ reads remained for further analysis (Table 1). Size distribution of reads was studied in each library. Size distribution of reads in each library showed two distinct peaks. One was observed at 20–23 nt corresponding to miRNAs and another at 32–35 nt representing piRNA like small RNAs (Figure 1). A total of 3.3 × 10^6^, 2.3 × 10^6^, 4.0 × 10^6^, 6.7 × 10^6^, 9.6 × 10^6^ and 2.8 × 10^6^ reads were found to lie between size range of 18-30 nt in LM, LF, PM, PF, AM and AF respectively. These sequences were mapped on to the known miRNA database for miRNA identification in various developmental stages of *An. stephensi* (Table 1). Unmatched sequences were mapped on the ncRNA database and coding sequences from different mosquito species (Table 1). The remaining unmatched sequences were used for novel miRNAs identification.

**Table 1:** **Composition of small RNA in deep sequenced libraries from larva male (LM), larva female (LF), pupa male (PM), pupa female (PF), adult male (AM) and adult female (AF) mosquito**

**Sample name**	**Raw reads**	**Reads post adaptor trimming**	**18-30 nt reads**	**Reads mapped to ncRNAs**	**Reads mapped to known miRNAs**	**Reads mapped to coding region of** ***Anopheles***	**Reads mapped to** ***An.stephensi*** **genome**
LM	3.5 × 10^6^	3.3 × 10^6^	3.3 × 10^6^	5.6 × 10^5^	1.1 × 10^4^	2.9 × 10^4^	2.9 × 10^5^
LF	2.7 × 10^6^	2.6 × 10^6^	2.3 × 10^6^	5.4 × 10^5^	6.3 × 10^4^	4.7 × 10^4^	1.8 × 10^5^
PM	1.6 × 10^7^	1.2 × 10^7^	4.0 × 10^6^	7.5 × 10^5^	6.2 × 10^5^	1.1 × 10^5^	4.0 × 10^5^
PF	1.9 × 10^7^	1.2 × 10^7^	6.7 × 10^6^	1.3 × 10^6^	8.3 × 10^5^	1.5 × 10^5^	7.0 × 10^5^
AM	2.5 × 10^7^	2.4 × 10^7^	9.6 × 10^6^	7.9 × 10^5^	2.6 × 10^6^	5.8 × 10^4^	9.9 × 10^5^
AF	5.4 × 10^6^	4.4 × 10^6^	2.8 × 10^6^	1.9 × 10^5^	7.8 × 10^5^	1.9 × 10^4^	5.2 × 10^5^
Total	7.2 × 10^7^	5.9 × 10^7^	2.8 × 10^7^	4.1 × 10^6^	4.9 × 10^6^	4.2 × 10^5^	3.1 × 10^6^

Complete repertoire of miRNAs in immature and mature stages of *An.stephensi* 18-30 nt length sequences in each of the libraries were mapped on to known miRNA sequences fetched from mirbase database. A total of 4.9×10^6^ reads from all six libraries mapped with 100% match with known miRNA sequences from eight different insect species (Table 1). After removing mapped redundancy, 111 total known miRNAs were identified across all stages of *An.stephensi.* Maximum and minimum number of known miRNAs were identified in AM (n = 109) and LF (n = 70) respectively (Table 2). Remaining stages LM, PM, PF, AF contained 85, 104, 106 and 101 known miRNAs respectively. Sixty nine miRNAs were expressed in all the stages of development of the mosquito. Remaining 42 were absent in one or more developmental stages of the mosquito (Table 2). Many miRNAs were absent in larval stage of mosquito although they showed expression with TPM >10 in other stages of development. Five miRNAs (miR-1891, miR-190-3p, miR-285, miR-988-3p and miR-989) were not expressed in both male and female larva. Whereas nine miRNAs (miR-bantam-5p, miR-137, miR-184b, miR-193, miR-71-5p, miR-929-5p, miR-980, miR-998 and miR-9c-3p) were found absent only in female larva. Out of these 111 miRNAs, three miRNAs namely miR-8-3p, miR-bantam-3p and miR-281-5p were found most abundant in developmental stages. MicroRNA-8-3p was the most abundant miRNA in larval stages of the mosquito while bantam-3p was most abundant in both male and female pupal stages of development. Most abundant miRNA in AM and AF was miR-281-5p and miR-bantam-3p respectively (Table 2). Differential expression of known miRNAs between the genders and during metamorphosis is described in following sections of the manuscript.

**Table 2:** **List of known miRNAs expressed and regulated across mosquito developmental stages**

**S. No.**	**miRNA**	**Length**	**Sequence**	**Tags Per Million (TPM)**					
				**LM**	**LF**	**PM**	**PF**	**AM**	**AF**
1	ast-bantam-3p	22	TGAGATCACTTTGAAAGCTGAT	2199.4	245.4	5410.0	6599.4	9907.7	25521.7
2	ast-bantam-5p	23	CCGGTTTTCATTTTCGATCTGAC	3.3		5.9	7.5	24.0	43.4
3	ast-let-7	21	TGAGGTAGTTGGTTGTATAGT	65.8	5.1	384.4	538.1	983.3	1301.1
4	ast-miR-100	22	AACCCGTAGATCCGAACTTGTG	254.1	30.9	2226.8	2173.4	3732.3	8010.1
5	ast-miR-1000	21	ATATTGTCCTGTCACAGCAGT	8.1	0.6	14.1	19.8	45.6	54.0
6	ast-miR-10-3p	23	CAAATTCGGTTCTAGAGAGGTTT	21.7	7.1	41.1	60.8	67.8	102.0
7	ast-miR-10-5p	22	ACCCTGTAGATCCGAATTTGTT	614.5	151.3	2437.9	3984.4	25072.2	9310.7
8	ast-miR-11	22	CATCACAGTCTGAGTTCTTGCT	218.1	23.8	370.2	574.2	626.4	1269.3
9	ast-miR-1174	22	TCAGATCTACTTCATACCCATG	18.0	2.0	7.9	7.4	6.9	20.6
10	ast-miR-1175-3p	21	TGAGATTCTACTTCTCCGACT	41.6	4.0	50.8	65.1	81.6	66.8
11	ast-miR-1175-5p	22	AAGTGGAGTAGTGGTCTCATCG	9.2		3.2	4.9	3.4	4.2
12	ast-miR-12	23	TGAGTATTACATCAGGTACTGGT	107.4	12.8	128.2	109.3	373.3	438.8
13	ast-miR-124	20	TAAGGCACGCGGTGAATGCC	1.5	0.3	7.2	9.7	40.1	45.1
14	ast-miR-125-3p	21	ACAAGTTTTGATCTCCGGTAT	1.1	0.3	3.6	4.9	3.5	11.0
15	ast-miR-125-5p	22	TCCCTGAGACCCTAACTTGTGA	10.3	1.1	66.7	80.3	228.9	194.0
16	ast-miR-133-3p	22	TTGGTCCCCTTCAACCAGCTGT	9.2	0.3	30.4	42.8	138.3	129.4
17	ast-miR-133-5p	20	AGCTGGTTGACATCGGGTCA				0.2	0.1	
18	ast-miR-137	22	TATTGCTTGAGAATACACGTAG	1.8		5.8	6.4	19.0	39.6
19	ast-miR-13a	22	CTCCTCAAAGGGTTGTGAAATG	0.4		0.4	0.6	1.3	1.3
20	ast-miR-13b-3p	23	TATCACAGCCATTTTGACGAGTT	44.1	9.6	135.1	190.7	138.4	124.9
21	ast-miR-13b-5p	22	TCGTAAAAATGGTTGTGCTGTG	3.7	2.3	6.9	14.3	5.5	18.6
22	ast-miR-1-3p	22	TGGAATGTAAAGAAGTATGGAG	19.5	2.8	243.3	259.6	428.2	197.9
23	ast-miR-14	22	TCAGTCTTTTTCTCTCTCCTAT	89.0	13.6	551.5	690.8	1779.2	4857.7
24	ast-miR-1-5p	18	TACTTCTTTACATTCCAT			0.2	0.4	0.2	
25	ast-miR-184a	22	TGGACGGAGAACTGATAAGGGC	1360.2	161.5	4415.3	4174.1	13173.6	16430.4
26	ast-miR-184b	22	TGGACGGAGAACTGATAAAGGA	22.8		12.0	151.6	76.9	115.5
27	ast-miR-1889	20	CACATTACAGATTGGGATTA			0.1	0.2	1.1	0.9
28	ast-mir-1890	20	TGAAATCTTTGATTAGGTCT	7.7	1.7	126.0	133.4	34.3	38.7
29	ast-miR-1891	22	TGAGGAGTTAATTTGCGTGTTT			0.1	0.3	129.2	85.4
30	ast-miR-190-3p	22	CCCAGGAATCAAACATATTATT			0.5	2.8	13.1	23.4
31	ast-miR-190-5p	24	AGATATGTTTGATATTCTTGGTTG	9.2	4.5	23.0	30.5	57.7	56.8
32	ast-miR-193	20	TACTGGCCTACTAAGTCCCA	0.7		8.4	15.9	0.3	1.1
33	ast-miR-210-3p	21	CTTGTGCGTGTGACAACGGCT	4.0	0.6	6.0	7.5	107.6	291.1
34	ast-miR-210-5p	21	AGCTGCTGACCACTGCACAAG			0.1		0.2	0.5
35	ast-miR-219	20	TGATTGTCCAAACGCAATTC			0.2	0.7	2.4	0.4
36	ast-miR-252-3p	21	CTGCTGCCCAAGTGCTTATCG	0.4		0.5	0.7	3.0	4.4
37	ast-miR-252-5p	22	CTAAGTACTAGTGCCGCAGGAG	60.7	5.7	128.4	166.9	950.6	1111.8
38	ast-mir-263a	22	AATGGCACTGGAAGAATTCACG	2272.9	575.0	831.3	887.0	1205.4	3564.4
39	ast-miR-263b-3p	21	TGGATCTTTTCGTGCCATCGT					0.1	
40	ast-miR-263b-5p	23	CTTGGCACTGGGAGAATTCACAG	5.5	2.3	17.3	25.8	133.4	137.4
41	ast-miR-275	22	TCAGGTACCTGAAGTAGCGCGC	135.0	11.9	286.9	451.9	341.0	711.3
42	ast-miR-276-3p	22	TAGGAACTTCATACCGTGCTCT	1118.3	136.9	2720.4	3464.7	3892.3	4702.5
43	ast-miR-2765	22	TGGTAACTCCACCACCGTTGGC	157.0	21.5	956.6	858.5	2.1	0.5
44	ast-miR-276-5p	22	AGCGAGGTATAGAGTTCCTACG	12.9	2.6	29.6	39.5	31.2	29.2
45	ast-miR-277	22	TAAATGCACTATCTGGTACGAC	36.8	6.0	605.0	657.4	3277.0	5776.7
46	ast-miR-278	21	TCGGTGGGACTTTCGTCCGTT	15.1	2.6	12.7	22.9	25.6	24.3
47	ast-miR-279	20	TGACTAGATCCACACTCATT	44.1	5.7	174.4	264.0	172.4	176.1
48	ast-miR-2796-3p	20	GTAGGCCGGCGGAAACTACT	2.6	0.6	3.2	4.3	20.1	25.7
49	ast-miR-2796-5p	22	AGGGGTTTCTTTCGGCCTCCAG			0.1	0.1	0.1	0.2
50	ast-mir-281-3p	22	TGTCATGGAATTGCTCTCTTTA	36.0	4.0	31.3	38.1	74.2	29.0
51	ast-miR-281-5p	22	AAGAGAGCTATCCGTCGACAGT	3986.2	233.8	4066.0	3725.7	14959.7	13394.8
52	ast-miR-282	22	TAGCCTCTTCTAGGCTTTGTCT	8.1	0.3	6.8	10.2	0.3	0.9
53	ast-miR-283	19	AAATATCAGCTGGTAATTC	1.8	0.6	2.4	2.7	6.8	5.5
54	ast-miR-285	22	TAGCACCATTCGAAATCAGTAC			51.4	14.2	313.7	4.2
55	ast-miR-286	25	TGACTAGACCGAACACTCGCGTCCT				0.1	0.1	
56	ast-miR-2944a-3p	22	TATCACAGTAGTTGTACTTTAA			0.1		0.2	
57	ast-miR-2944a-5p	22	GAAGGAACTTCTGCTGTGATCT			0.4	1.3	0.8	0.4
58	ast-miR-2944b-3p	22	TATCACAGCAGTAGTTACCTGA				0.1	0.0	
59	ast-miR-2944b-5p	23	GAAGGAACTCCCGGTGTGATATA			0.1	0.1	0.1	0.2
60	ast-miR-2945	19	TGACTAGAGGCAGACTCGT	0.4		1.6	2.5	3.5	4.7
61	ast-miR-2a-3p	23	TATCACAGCCAGCTTTGAAGAGC	110.3	13.3	280.0	375.7	475.8	601.6
62	ast-miR-2a-5p	21	ACTCTCAAAGTGGTTGTGAAA	14.0	2.0	79.0	35.4	8.8	36.5
63	ast-miR-2b	24	TATCACAGCCAGCTTTGATGAGCT	51.5	8.5	139.2	307.4	295.8	225.8
64	ast-miR-2c	18	TCACAGCCAGCTTTGATG	7.4		0.2	0.8	4.3	
65	ast-miR-305-3p	22	CGGCACATGTTGGAGTACACTT	5.1	0.3	8.8	8.8	20.8	21.9
66	ast-miR-305-5p	21	ATTGTACTTCATCAGGTGCTC	95.2	9.9	189.8	288.3	168.4	277.6
67	ast-miR-306	22	TCAGGTACTGGATGACTCTCAG	410.0	39.4	556.8	792.3	845.3	2160.1
68	ast-miR-307-3p	20	TCACAACCTCCTTGAGTGAG			0.3	0.6	1.2	1.5
69	ast-miR-307-5p	20	ACTCACTCAACCTGGGTGTG			0.1	0.1	0.1	0.2
70	ast-miR-308	18	AATCACAGGAGTATACTG	32.4	2.6	22.7	30.4	13.0	24.5
71	ast-miR-309	22	TCACTGGGCAAAGTTTGTCGCA				0.1		
72	ast-miR-31	21	TGGCAAGATGTTGGCATAGCT	14.0	2.8	48.6	53.6	104.0	63.7
73	ast-miR-315	23	TTTTGATTGTTGCTCAGAAAGCC	129.1	34.6	75.8	129.8	382.3	980.6
74	ast-miR-317	25	TGAACACATCTGGTGGTATCTCAGT	111.8	17.6	60.2	47.6	480.5	1900.9
75	ast-miR-33	21	GTGCATTGTAGTTGCATTGCA		0.3	0.2	0.2	0.6	0.5
76	ast-miR-34	23	TGGCAGTGTGGTTAGCTGGTTGT	18.0	2.8	8.5	8.0	789.4	657.3
77	ast-miR-375	22	TTTGTTCGTTTGGCTCGAGTTA	72.1	6.0	8.5	11.6	55.5	145.1
78	ast-miR-7	21	TGGAAGACTAGTGATTTTGTT	8.5	2.3	1.9	2.4	13.6	2.9
79	ast-miR-71-3p	22	TCTCACTACCTTGTCTTTCATG	26.5	2.8	64.8	80.9	97.0	98.0
80	ast-miR-71-5p	22	AGAAAGACATGGGTAGTGAGAT	1.1		5.6	5.6	15.3	8.4
81	ast-miR-79-3p	23	ATAAAGCTAGATTACCAAAGCAT	0.4	0.6	1.5	3.0	2.3	3.8
82	ast-miR-79-5p	23	GCTTTGGCGCTTTAGCTGTATGA			0.3	0.6	0.2	0.5
83	ast-miR-8-3p	23	TAATACTGTCAGGTAAAGATGTC	4190.3	577.3	3906.2	5280.5	8065.6	24812.4
84	ast-miR-8-5p	22	CATCTTACCGGGCAGCATTAGA	130.2	13.6	230.3	285.7	901.1	2144.5
85	ast-miR-87	19	GTGAGCAAATATTCAGGTG	0.4		0.2	0.2	1.3	1.3
86	ast-miR-927-3p	22	CAAAGCGTTTGGATTCTGAAAC	4.0	0.9	11.8	15.3	50.7	126.7
87	ast-miR-927-5p	22	TTTAGAATTCCTACGCTTTACC	22.8	7.1	146.7	97.7	866.4	934.0
88	ast-miR-929-3p	20	TCCCTAACGGAGTCAGATTG					0.2	0.4
89	ast-miR-929-5p	21	AAATTGACTCTAGTAGGGAGT	0.4		4.2	4.3	13.5	15.5
90	ast-miR-92a	20	TATTGCACTTGTCCCGGCCT	36.4	1.7	33.1	21.3	29.7	91.3
91	ast-miR-92b	22	AATTGCACTTGTCCCGGCCTGC	192.0	11.9	283.7	339.2	147.6	218.3
92	ast-miR-932	23	TCAATTCCGTAGTGCATTGCAGT	4.8	0.9	22.7	28.1	55.7	80.9
93	ast-miR-957	22	TGAAACCGTCCAAAACTGAGGC	68.4	24.4	268.1	366.7	898.2	819.6
94	ast-miR-965	22	TAAGCGTATAGCTTTTCCCATT	1.1		2.0	2.8	0.8	2.0
95	ast-miR-970-3p	21	TCATAAGACACACGCGGCTAT	43.4	3.4	75.9	131.4	313.4	453.4
96	ast-miR-980	19	TAGCTGCCTAGTGAAGGGC	0.4		1.6	1.9	13.3	37.8
97	ast-miR-981	22	TTCGTTGTCGACGAAACCTGCA	4.8	0.9	8.2	17.3	63.0	49.1
98	ast-miR-988-3p	22	CCCCTTGTTGCAAACCTCACGC			2.5	8.3	12.1	27.6
99	ast-miR-988-5p	21	GTGTGCTTTGTGACAATGAGA			0.1		0.2	
100	ast-miR-989	21	TGTGATGTGACGTAGTGGTAC			4.5	2.1	0.5	55.1
101	ast-miR-993-3p	24	GAAGCTCGTTTCTATAGAGGTATC	4.4	2.3	6.7	9.2	14.7	28.5
102	ast-miR-993-5p	22	TACCCTGTAGTTCCGGGCTTTT	1.1	0.9	3.4	6.7	10.6	15.7
103	ast-miR-996	20	TGACTAGATTACATGCTCGT	68.8	6.5	149.5	199.4	175.1	315.8
104	ast-miR-998	21	TAGCACCATGAGATTCAGCTC	7.4		14.3	10.1	33.9	38.3
105	ast-miR-999	22	TGTTAACTGTAAGACTGTGTCT	210.0	24.9	440.1	609.1	2221.6	3492.3
106	ast-miR-9a	23	TCTTTGGTTATCTAGCTGTATGA	2025.1	406.1	1594.9	2663.9	321.4	1049.7
107	ast-miR-9c-3p	22	TAAAGCTTTAGTACCAGAGGTC	2.6		2.1	3.1	3.9	11.1
108	ast-miR-9c-5p	22	TCTTTGGTATTCTAGCTGTAGA	840.6	92.1	669.4	1056.2	366.8	1565.7
109	ast-miR-iab-4-3p	24	CGGTATACCTTCAGTATACGTAAC				0.1		
110	ast-miR-iab-4-5p	22	ACGTATACTGAATGTATCCTGA	3.3	0.3	2.3	2.6	1.7	2.2
111	ast-miR-iab-8-5p	20	TTACGTATACTGAAGGTATA			0.1	0.2	0.2	0.2

Several novel miRNAs were identified using high throughput deep sequencing data in adult mosquito. Novel miRNAs identified in adult female mosquito were reported previously [9]. In this study, we focused on identification of novel miRNAs in adult male mosquito. Their expression was then checked in other stages of mosquito development (Table 3). Fourteen small RNA sequences folded into a perfect hairpin structure with folding energy < −20 kcal/mol (Additional file 1: Figure S1). Seven of these were present on 5’ arm of the precursor and hence were annotated as miR-5p whereas rest seven sequences were present on 3’arm and were annotated as miR-3p. Nomenclature of these novel miRNAs was given in a specific order (Table 3). Novel miRNA sequences were blasted against known miRNAs in mirBase database. These sequences did not show similarity with any of the known miRNAs indicating towards identification of novel class of miRNAs in mosquito.

**Table 3:** **List of Novel miRNAs expressed and regulated across mosquito developmental stages**

**S. No**	**Length**	**Mature miRNA sequence**	**Pre-miRNA sequence**	**Novel miRNA**	**Tags Per Million (TPM)**					
					**LM**	**LF**	**PM**	**PF**	**AM**	**AF**
1	20	AGTCATTAATGATATTAGAC	AUUUAUGGGAAAUAAUCUCUUUAAUCAGGCUUUAUAGUCAUUAAUGAUAUUAGACUGCAAUUCUAAAGGA AUAAUUUAAUUUAUUAAGGCUUAAGAAUUAAAAGAUUAAUACAAAUAUUUUCA	ast-Novel-1-3p	0.37	0.00	0.06	0.05	0.08	0.00
2	21	ATAATTTAATTTATTAAGGCT	AUUUAUGGGAAAUAAUCUCUUUAAUCAGGCUUUAUAGUCAUUAAUGAUAUUAGACUGCAAUUCUAAAGGA AUAAUUUAAUUUAUUAAGGCUUAAGAAUUAAAAGAUUAAUACAAAUAUUUUCA	ast-Novel-1-5p			0.18	0.16	2.84	0.18
3	23	GTATTCAATTTGTATATCGTCGT	GUUGGACCUUACUUAAAUUUGUAUUCAAUUUGUAUAUCGUCGUCAUCAGAAUAUAUUAUAAGAUUAAUAAUUUUCUAAAUAUUUUAUUAAAUAAUAUGUCAGGUCAAGGUGCAGUUUAUGUUUAAGUAG	ast-Novel-2-3p			0.18	0.10	0.08	
4	19	ATTGATAATCCACGTTGGA	GUUGGACCUUACUUAAAUUUGUAUUCAAUUUGUAUAUCGUCGUCAUCAGAAUAUAUUAUAAGAUUAAUAAUUUUCUAAAUAUUUUAUUAAAUAAUAUGUCAGGUCAAGGUGCAGUUUAUGUUUAAGUAG	ast-Novel-2-5p	0.37		0.54	0.16	0.11	
5	24	TTAAGATTGGTATGATTAGCGTCT	UUUCCUUUAAGAUUGGUAUGAUUAGCGUCUUGUUUAGCAGAAACUAAUCGUACUCCUUUU	ast-Novel-3-3p				0.16	0.12	0.24
6	24	AAACTAATCGTACTCCTTTTGATT	UUUCCUUUAAGAUUGGUAUGAUUAGCGUCUUGUUUAGCAGAAACUAAUCGUACUCCUUUU	ast-Novel-3-5p					0.16	
7	18	AATGGGTAGTCGAAGATT	AAUUGCUCAUGGUUUAUGUUCAUCUGGAUUAUUUUGUUUAGCUAAUAUUUCUUACGAACGAAUGGGUAGUCGAAGAUUAUUAAUUAAUAA	ast-Novel-4-3p			6.00	0.47	0.16	
8	22	AATTGCTCATGGTTTATGTTCA	AAUUGCUCAUGGUUUAUGUUCAUCUGGAUUAUUUUGUUUAGCUAAUAUUUCUUACGAACGAAUGGGUAGUCGAAGAUUAUUAAUUAAUAA	ast-Novel-4-5p					0.3	
9	21	CGTAGTTTCTACATTAGGAGT	UUUUACUUUUUUUGUAGGAUCAAUAUGAUUUAUACCCGUAGUUUCUACAUUAGGAGUAAU	ast-Novel-5-3p			0.12	0.10	0.04	
10	21	ACTTTTTTTGTAGGATCAATA	UUUUACUUUUUUUGUAGGAUCAAUAUGAUUUAUACCCGUAGUUUCUACAUUAGGAGUAAU	ast-Novel-5-5p			0.60	0.42	0.56	0.18
11	25	TTTCGGATATGAATCAAAGTAATTT	ACCACUUAAAUUUCGGAUAUGAAUCAAAGUAAUUUUCAUCAAUUCCCUCUACCCUCAUGAGCGAGUGAUGUAAUGUACUCUCGUUCUGGACGAUUUUCAACCACUUAAAU	ast-Novel-6-3p	100.76	44.78	95.03	141.73	135.20	796.74
12	21	CGTTCTGGACGATTTTCAACC	ACCACUUAAAUUUCGGAUAUGAAUCAAAGUAAUUUUCAUCAAUUCCCUCUACCCUCAUGAGCGAGUGAUGUAAUGUACUCUCGUUCUGGACGAUUUUCAACCACUUAAAU	ast-Novel-6-5p	0.37				0.6	
13	23	AGCCGAACCAGCAGTACGAGTTT	GACCCGACCGAGCCGAACCAGCAGUACGAGUUUUAACGCACGUUUCUUCGACUGGGAGCGUGGCGUCUCCUGUAACGCGGCUACUCGUGG	ast-Novel-7-3p					0.04	
14	22	CGTCTCCTGTAACGCGGCTACT	GACCCGACCGAGCCGAACCAGCAGUACGAGUUUUAACGCACGUUUCUUCGACUGGGAGCGUGGCGUCUCCUGUAACGCGGCUACUCGUGG	ast-Novel-7-5p		0.28	0.30	0.31	0.04	0.18

### Validation of known miRNAs by Northern hybridization

**Figure 2:**
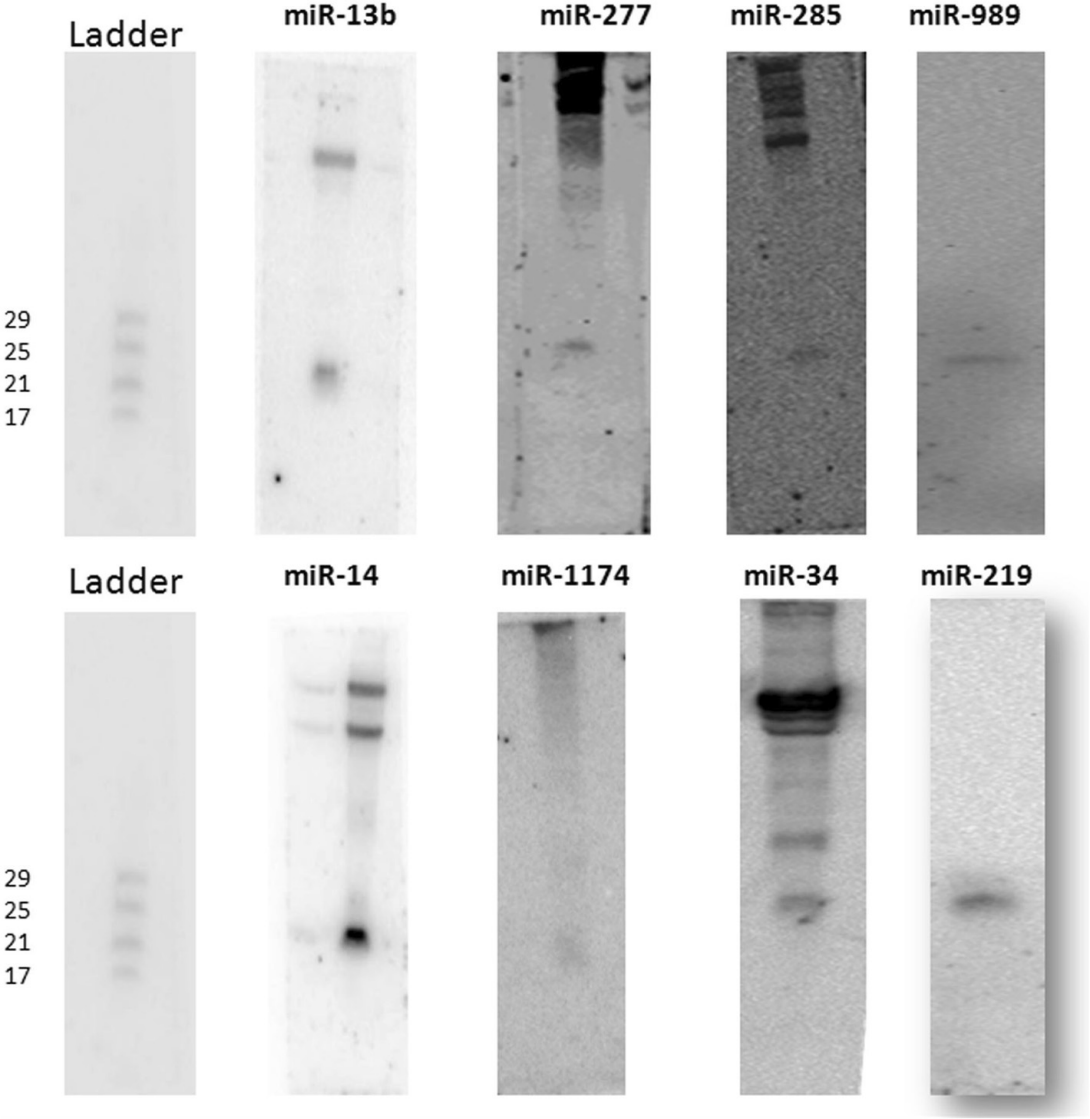
**Northern hybridization based detection of mosquito miRNAs.** 10ug total RNA from adult female was used for detection of **(A)** miR-13b, **(B)** miR-277, **(C)** miR-285, **(D)** miR-989, **(E)** miR-14, **(F)** miR-1174, **(G)** miR-34 and **(H)** miR-219 via DIG based Northern hybridization. Ladder was run along with total RNA with oligos ranging from 17 nt to 29 nt.

MicroRNAs identified by small RNA sequencing was verified by northern hybridization. Total RNA from sugar fed female mosquito was probed using locked nucleic acid (LNA) probes (Exiqon) for representative miRNAs. Expression of eight miRNAs namely miR-277, miR-14, miR-34, miR-285, miR-13b-3p, miR-989, miR-1174 and mir-219 was validated in *An.stephensi* (Figure 2). Out of these eight miRNAs, miR-277 and miR-14 were highly abundant in female mosquito with reads more than 2×10^4^. Hence, these two miRNAs showed distinct and specific band in northern hybridization. Two MicroRNA miR-285 and miR-219 were low abundance miRNAs with reads less than 25 in female mosquito. Nevertheless, such low expressing miRNAs was detectable by specific LNA based probes in the mosquito (Figure 2) thereby validating the sensitivity of this assay.

### Differential expression of miRNAs across different stages of mosquito development

**Figure 3:**
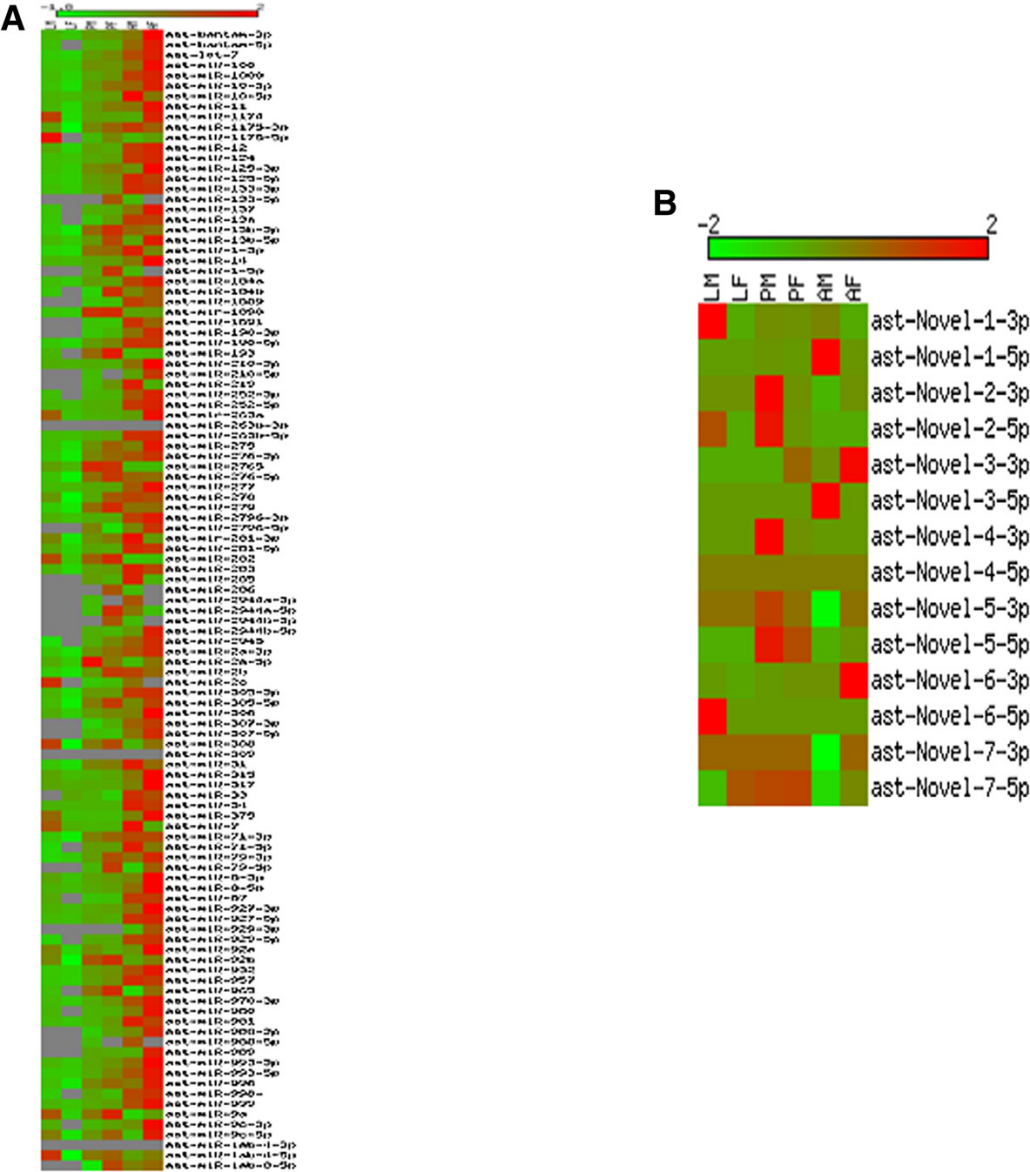
**Heat map of miRNAs differentially expressed across developmental stages.** Expression profiles of **(A)** known miRNAs and **(B)** Novel miRNAs in small RNA libraries prepared from larva male (LM), larva female (LF), pupa male (PM), pupa female (PF), adult male (AM) and adult female (AF) mosquito. Colour gradation from light green to dark red represents relative increase in miRNA expression.

To study differential expression of miRNAs, tags per million (TPM) of individual miRNAs was calculated in all the six libraries. MicroRNAs with TPM >10 in any of the developmental stages were subjected to EdgeR analysis. Regulation of miRNAs was studied between two developmental stages and miRNAs showing fold change >2 with P value ≤ 0.05 were classified as differentially expressed. Heat map of all known miRNAs is shown in Figure 3. A total of 36 miRNAs were found differentially expressed between different stages of mosquito development.

#### Gender regulated microRNAs

We studied difference in miRNAs expression between both genders during larval, pupa and adult mosquito stages. Minimum number of miRNA differences was identified during larval stages as it is the early stage of mosquito life cycle. Only two miRNAs namely miR-184b and miR-1175-5p were significantly down-regulated in female larvae when compared with male larvae (Additional file 2: Figure S2). Both genders during pupa stage showed difference in the expression of three miRNAs. MicroRNA-285 was down-regulated whereas miR-190-3p and miR-184b were up-regulated in female pupa as compared to male pupa stage (Additional file 2: Figure S2). Maximum differences in miRNA expression between genders were observed during adult stages of the mosquito. Only one miRNA miR-989 was found significantly up-regulated in adult female mosquito when compared to male mosquito. Whereas, five miRNAs namely miR-2c, miR-285, miR-219, miR-7 and miR-2765 were found down-regulated in female compared with male mosquito (Additional file 2: Figure S2).

#### MicroRNA regulation during metamorphosis of male mosquito from larval to pupa and to adult stages

MicroRNA regulation was observed during transition of male mosquito from larva to pupa stage and during metamorphosis from pupa to adult stage of mosquito life cycle. Many miRNAs were found differentially expressed during these two transition phases. Seven miRNAs (miR-2c, miR-375, miR-7, miR-1175-5p, miR-263a, miR-1174 and miR-34) were down-regulated whereas cluster of seven miRNAs (miR-100, miR-193, miR-1-3p, miR-1890, miR-277, miR-989 and miR-285) were up-regulated in pupa stage when compared to the larval male mosquito (Additional file 3: Figure S3). Metamorphosis from pupa to adult stage also resulted in differential expression of miRNAs. We identified seven miRNAs (miR-10-5p, miR-219, miR-2c, miR-210-3p, miR-190-3p, miR-34 and miR-1891) up-regulated in male adult mosquito when compared to pupal stage (Additional file 3: Figure S3). Transition to adult mosquito resulted in down-regulation of eight miRNAs namely miR-2765, miR-193, miR-282, miR-989, miR-2a-5p, miR-9a, miR-1890 and miR-965 (Additional file 3: Figure S3). Out of these regulated miRNAs, five miRNAs are of significant importance as they showed differential expression during larval to pupa as well as during pupa to adult metamorphosis. Two microRNAs, miR-2c and miR-34 were down-regulated from larval to pupa transition whereas its expression increases again in adult stages. Three miRNAs, miR-193, miR-1890 and miR-989 were up-regulated in pupa stage. Their expression down-regulates in adult male mosquito when compared to its pupa stage (Additional file 3: Figure S3).

#### MicroRNA regulation during metamorphosis of female mosquito from larval to pupa and to adult stages

Regulation of miRNAs was also observed during metamorphosis of female mosquito from larva to pupa and to adult stages of development. Eight miRNAs (miR-1-3p, miR-let-7, miR-277, miR-133-3p, miR-285, miR-193, miR-998 and miR-184b) were up-regulated in pupa female when compared to larva female mosquito (Additional file 4: Figure S4). Another set of eight miRNAs (miR-7, miR-263a, miR-375, miR-317, miR-34, miR-1174, miR-315 and miR-993-3p) were found down-regulated in pupa female compared with larval female mosquito (Additional file 4: Figure S4). Metamorphosis of female pupa to adult also resulted in differential expression of miRNAs. We identified eight miRNAs (miR-927-5p, miR-375, miR-980, miR-989, miR-210-3p, miR-317, miR-34 and miR-1891) up-regulated in adult female mosquito compared to pupa stage (Additional file 4: Figure S4). A set of eight miRNAs (miR-2765, miR-2c, miR-1-5p, miR-193, miR-282, miR-2944a-5p, miR-285 and miR-9a) were down-regulated in adult mosquito when compared with pupa female mosquito (Additional file 4: Figure S4). Five miRNAs were differentially expressed in all developmental stages of female mosquito. Two miRNAs, miR-285 and miR-193 were up-regulated in pupa stages whereas gets down-regulated in adult stages. Three miRNAs namely miR-375, miR-317 and miR-34 were down-regulated in pupa female whereas adult stage resulted in up-regulation of these miRNAs when compared to pupa stage (Additional file 4: Figure S4).

### Validation of miRNA expression profiling by real-time PCR

**Figure 4:**
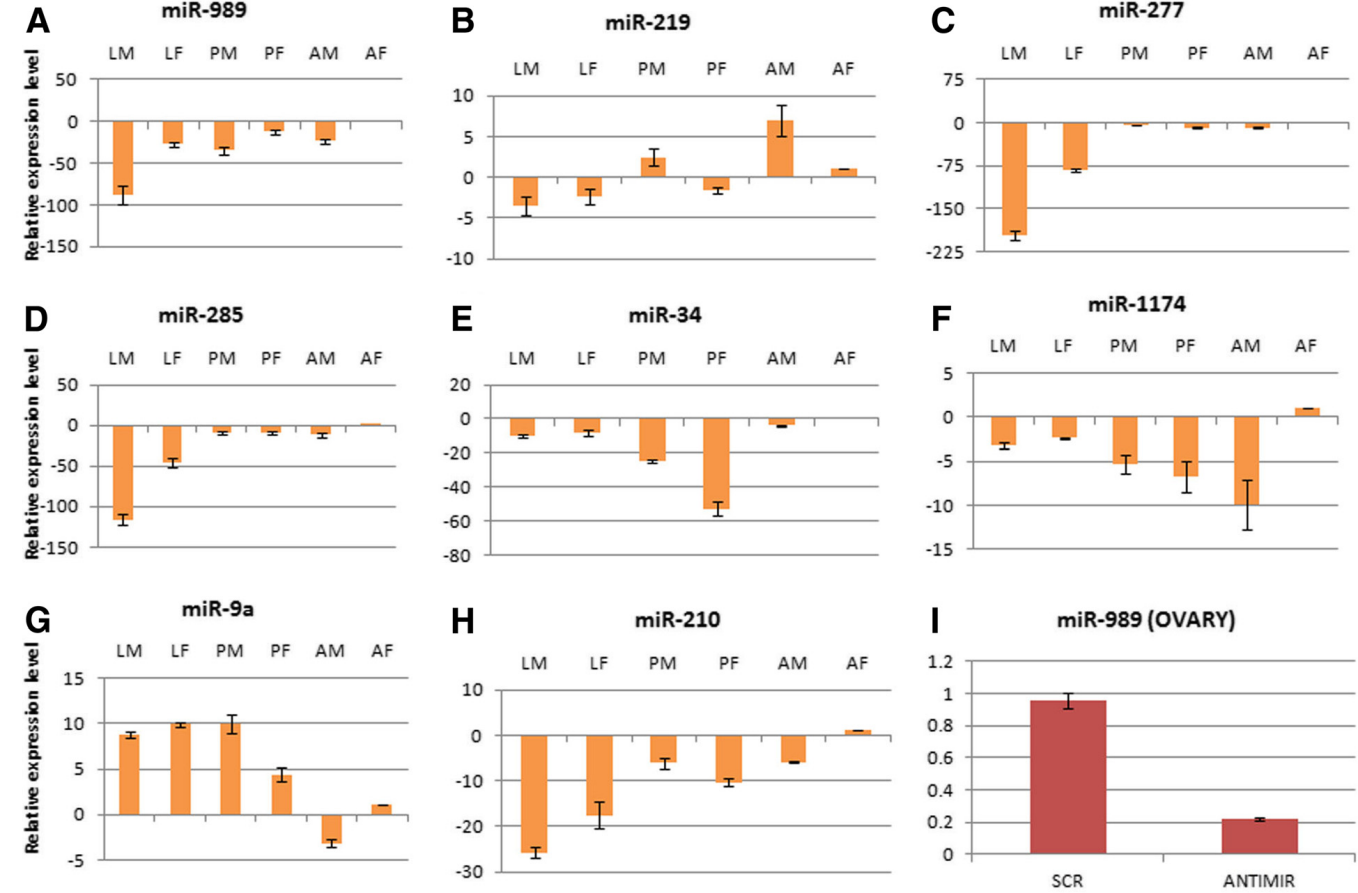
**Expression profiling of regulated miRNAs by Real time PCR. (A)** miR-989, **(B)** miR-219, **(C)** miR-277, **(D)** miR-285, **(E)** miR-34, **(F)** miR-1174, **(G)** miR-9a and **(H)** miR-210 were profiled in larva male (LM), larva female (LF), pupa male (PM), pupa female (PF), adult male (AM) and adult female (AF) mosquito. Y axis depicts fold change in miRNA expression in samples compared with adult female mosquito (AF), fold change in which was taken as 1. **(I)** Fold change in miRNA-989 expression in ovaries from scrambled (SCR) and antagomir (ANTIMIR) injected mosquitoes. Y axis depicts fold change in miRNA expression in samples compared with PBS injected ovaries, fold change in which was taken as 1. 5.8 s RNA was taken as an endogenous control.

Differential expression of miRNAs across immature and mature stages in both male and female mosquitoes was validated by qRT-PCR. Two miRNAs, miR-989 and miR-219 were up-regulated in adult female and adult male mosquito respectively when compared to other stages of development (Figure 4A and B). Significant down-regulation was observed for miR-277, miR-210 and miR-285 in larval stages compared to pupa and adult stages of mosquito (Figure 4C, D and H). MicroRNA-34 was down-regulated in male and female pupa compared to larva and adult mosquito (Figure 4E). MicroRNA-1174 expression was down-regulated in both genders of pupa stage and male mosquito (Figure 4F). MicroRNA -9a was the only miRNA that showed up-regulated expression in larva and pupa stages when compared to adult mosquito (Figure 4G). Similar expression pattern of miRNAs was observed in small RNAs sequencing and real time data analysis, thereby validating our analysis.

### MicroRNA target prediction using RNA hybrid and degradome sequencing

Regulation of gene expression is brought about by binding of miRNAs on 3’UTR sequence of the target genes and in silico analysis for identifying miRNA binding regions could be employed for better validation [25]. In the present study, we employed bioinformatic analysis and *in vivo* assays to identify targets of regulated miRNAs. Targets binding to miRNAs were predicted first by identifying miRNAs seed binding sites on 3’UTR of *An. stephensi* genes using RNAhybrid. Further, degree of binding complementarity determines the mechanism of target regulation, either by mRNA cleavage or by translational repression. As ovary is an important organ for insect reproduction, we aimed to identify mRNAs cleaved by miRNAs in ovary tissue of blood fed female mosquito by degradome sequencing.

#### Target prediction of differentially expressed miRNAs and enriched pathway analysis

Targets of miRNAs regulated in gender-specific manner and during metamorphosis from larva to pupa and to adult stages in both genders were predicted using RNAhybrid (P value < 0.05). mRNA targets were predicted for 36 miRNAs that were regulated during different stages of mosquito development (as described above) (Additional file 5: Table S1). Maximum number of targets were identified for miR-34 (n = 524) whereas minimum targets were predicted for miR-190-3p (n = 24). Orthologs of all the mRNA targets known in *An.gambiae* were retrieved from vector-base. MicroRNAs were divided into five clusters depending upon developmental stage at which they were found differentially expressed. MicroRNAs that showed gender differential expression during adult stage were grouped as a cluster. MicroRNAs regulated between the developmental stages in both genders were analysed separately (Table 4). Orthologs of target genes known in *An. gambiae* were retrieved from vector base and analysed using functional annotation clustering tool of DAVID resource. Analysis was carried out using *An.gambiae* gene IDS as *An.stephensi* database is not linked to the DAVID resource. Annotation term groups playing more enriched role in present study were identified. Targets of miRNAs showing gender differential expression were analysed which resulted in identification of five enriched annotation clusters. These clusters were involved in cellular polysaccharide biosynthetic process, chaperone, hexose metabolic pathway, heme binding and phagocytotic pathways (Table 4). Targets of miRNAs regulated from larva to pupa stage in male mosquito were enriched in genes functional in phagocytosis and membrane organization, polysaccharide metabolic process, protease activity, peptidase activity and nuclear transport. Female larva to pupa metamorphosis involved genes functional in enriched pathways such as endocytosis, polysaccharide metabolic process, cell-redox homeostasis, peptidase and amino acid metabolic pathway (Table 4). Transition of pupa to adult male mosquito is mediated by enriched pathways such as gelsolin–actin binding protein, polysaccharide biosynthetic process, zinc finger, vesicle mediated transport, protein biosynthesis, translation, vitamin and cofactor binding, nucleoside binding, protein catabolic process and insect pheromone /odorant binding protein phBP (Table 4). In female mosquitoes, pathways functional in phagocytosis, cellular carbohydrate biosynthetic process, protein biosynthesis, vitamin binding, cyclin, golgi membrane, ATP binding, hydrolase activity and transcription were enriched during pupa to adult metamorphosis (Table 4).

**Table 4:** **List of enriched pathways functional during different stages of mosquito development**

(I)	**miRNAs differentially expressed in male and female adult mosquitoes**	
	Down-regulated miRNAs in females: miR-2c, miR-285, miR-219, miR-7 and miR-2765	
	Up-regulated miRNAs in females: miR-989	
	**Significant clusters identified**	**Enrichment score**
1	Cellular polysaccharide biosynthetic process	2.1
2	Chaperone	1.8
3	Hexose metabolic pathway	1.3
4	Heme binding	1.1
5	Phagocytotic pathways	1.09
(II)	**miRNAs regulated from larval to pupa transition of male mosquito**	
	Down-regulated miRNAs in pupa male: miR-2c, miR-375, miR-7, miR-1175, miR-263a, miR-1174 and miR-34	
	Up-regulated miRNAs in pupa male: miR-100, miR-193, miR-1-3p, miR-1890, miR-277, miR-989 and miR-285	
	**Significant clusters identified**	**Enrichment score**
1	phagocytosis and membrane organization	2.6
2	polysaccharide metabolic process	2.1
3	protease activity	1.9
4	peptidase activity	1.6
5	nuclear transport	1.1
(III)	**miRNAs regulated from transition of pupa to adult male mosquito**	
	Down-regulated miRNAs in adult male: miR-2765, miR-193, miR-282, miR-989, miR-2a-5p, miR-9a, miR-1890 and miR-965	
	Up-regulated miRNAs in adult male: miR-10-5p, miR-219, miR-2c, miR-210-3p, miR-190-3p, miR-34 and miR-1891	
	**Significant clusters identified**	**Enrichment score**
1	gelsolin –actin binding protein	2.2
2	polysaccharide biosynthetic process	2
3	zinc finger	1.8
4	vesicle mediated transport	1.7
5	protein biosynthesis	1.6
6	translation	1.5
7	vitamin and cofactor binding	1.4
8	nucleoside binding	1.3
9	protein catabolic process	1.2
10	insect pheromone/odorant binding protein phBP	1.1
(IV)	**miRNAs regulated from larval to pupa transition of female mosquito**	
	Down-regulated miRNAs in pupa female: miR-7, miR-263a, miR-375, miR-317, miR-34, miR-1174, miR-315 and miR-993-3p	
	Up-regulated miRNAs in pupa female : miR-1-3p, miR-let-7, miR-277, miR-133-3p, miR-285, miR-193, miR-998 and miR-184b	
	**Significant clusters identified**	**Enrichment score**
1	endocytosis	3
2	polysaccharide metabolic process	1.9
3	cell-redox homeostasis	1.7
4	peptidase	1.5
5	amino acid metabolic pathway	1.4
(v)	**miRNAs regulated from transition of pupa to adult female mosquito**	
	Down-regulated miRNAs in adult female: miR-2765, miR-2c, miR-1-5p, miR-193, miR-282, miR-2944a-5p, miR-285 and miR-9a	
	Up-regulated miRNAs in adult female : miR-927-5p, miR-375, miR-980, miR-989, miR-210-3p, miR-317, miR-34 and miR-1891	
	**Significant clusters identified**	**Enrichment score**
1	phagocytosis	2.4
2	cellular carbohydrate biosynthetic process	2.1
3	protein biosynthesis	1.6
4	vitamin binding	1.5
5	golgi membrane	1.1
6	ATP binding	1.1
7	hydrolase activity	1.1
8	transcription	1.1

These pathways were identified using NIH DAVID functional annotation clustering tool with E score =0.1.

#### Target identification using antagomirs and degradome sequencing

Loss of function strategy was employed for identification of miRNA targets in ovary of female mosquito. PBS, scrambled RNA and miRNA specific antagomirs were Nano-injected in female mosquito. RNA was extracted and subjected to degradome sequencing. Degradome libraries were constructed from ovary tissue of PBS, scrambled RNA and miR-989 antagomir injected mosquitoes respectively dissected at 24 hours post blood feeding. Analysis of data from PBS injection revealed cleaved products of several miRNAs identified in our study (Data not shown). We restricted our analysis to those significantly regulated miRNAs between the genders, namely, miR-2c, miR-285, miR-219, miR-7, miR-989 and miR-2765. These miRNAs showed distinct regulation in their expression pattern between the two genders at their reproductively active mature stages. Such difference in expression between the genders highlights their role in reproductive processes. mRNA targets of these miRNAs were identified by sequencing the degraded mRNA due to miRNA cleavage using PARE sequencing. Cleaved targets were identified for four miRNAs (miR-285, miR-219, miR-989 and miR-7). Twenty nine mRNAs cleaved by these miRNAs as visualised by target plots were identified in ovary of female mosquito. mRNAs (n = 6) were cleaved by miR-219 and miR-7 whereas miR-285 cleaved only one mRNA in ovary tissue of female mosquito (Table 4). Maximum number if targets were found to be cleaved by miR-989 (n = 16).

In an attempt to validate these targets and to understand the role of miR-989 in mosquito reproduction, expression of miR-989 was knocked down by injecting miR-989 specific antagomir in female mosquito. Four fold decreases in miRNA expression was observed in antagomir injected tissue compared to PBS and scrambled injected ovaries (Figure 4I). Of the sixteen mRNAs that were found to be cleaved in the ovary of PBS injected mosquitoes, ten mRNAs were found regulated in miR-989 antagomir injected ovaries (Table 5). Four of the cleaved targets (ASTE002227, ASTE008381, ASTE003701 and ASTE005749) were also predicted as miR-989 targets by RNAhybrid (Table 5).

**Table 5:** **List of miRNA targets identified by degradome sequencing in ovary tissue of female mosquito**

**S. No.**	**miRNA**	**Target**	**OVARY_PBS (Category)**	**OVARY_SCR (Category)**	**OVARY_989 (Category)**	**GO term name**	**Cleavage position**	**Ortholog in** ***An. gambiae***
1	miR-989	ASTE002227*	yes (2)	yes (4)	yes (2)	protein binding	500	AGAP003558
2	miR-989	ASTE014169	yes (0)	yes (0)	yes (0)		151	
3	miR-989	ASTE004090	yes (3)	yes (3)	yes (4)	signal transduction	3429	AGAP002261
4	miR-989	ASTE010155	yes (0)			DNA binding	840	AGAP003982
5	miR-989	ASTE008099	yes (4)			protein binding	553	AGAP003439
6	miR-989	ASTE003651	yes (4)			aminoacyl-tRNA ligase activity	1680	AGAP004708
7	miR-989	ASTE007120	yes (4)	yes (2)	yes (2)		2092 and 1931	AGAP000879
8	miR-989	ASTE014120	yes (2)	yes (4)	yes (2)		275	
9	miR-989	ASTE010290	yes (4)	yes (2)	yes (2)		1682	AGAP005504
10	miR-989	ASTE002911	yes (2)	yes (4)	yes (2)	nucleic acid binding	731	AGAP004361
11	miR-989	ASTE008381*	yes (4)		yes (2)		984	AGAP010075
12	miR-989	ASTE004431	yes (4)			proteolysis	1824	AGAP007280
13	miR-989	ASTE003701*	yes (4)				795	AGAP010226
14	miR-989	ASTE011654	yes (2)	yes (3)	yes (2)		2304	
15	miR-989	ASTE005749*	yes (4)				2347	AGAP001150
16	miR-989	ASTE003996	yes (2)		yes (4)	integral component of membrane	1681	AGAP004486
17	miR-219	ASTE006120	yes (4)				1650	AGAP004892
18	miR-219	ASTE006196	yes (4)			proteolysis	4239	AGAP006203
19	miR-219	ASTE011346	yes (4)				1764	AGAP004892
20	miR-219	ASTE007196	yes (4)				1442	AGAP001357
21	miR-219	ASTE002692	yes (4)			GTP binding	1763	AGAP009441
22	miR-219	ASTE009304	yes (4)			protein binding	1268	AGAP011439
23	miR-285	ASTE005579	yes (2)			acid phosphatase activity	337	AGAP002387
24	miR-7	ASTE008161	yes (4)				428	AGAP010789
25	miR-7	ASTE001260	yes (4)				677	AGAP008905
26	miR-7	ASTE008160	yes (4)				383	AGAP010789
27	miR-7	ASTE010939	yes (4)				2608	AGAP012493
28	miR-7	ASTE002046	yes (4)			ATP binding	211	AGAP002996
29	miR-7	ASTE003640	yes (2)			metabolic process	1342	AGAP004721

*Targets predicted by RNAhybrid.

**Figure 5:**
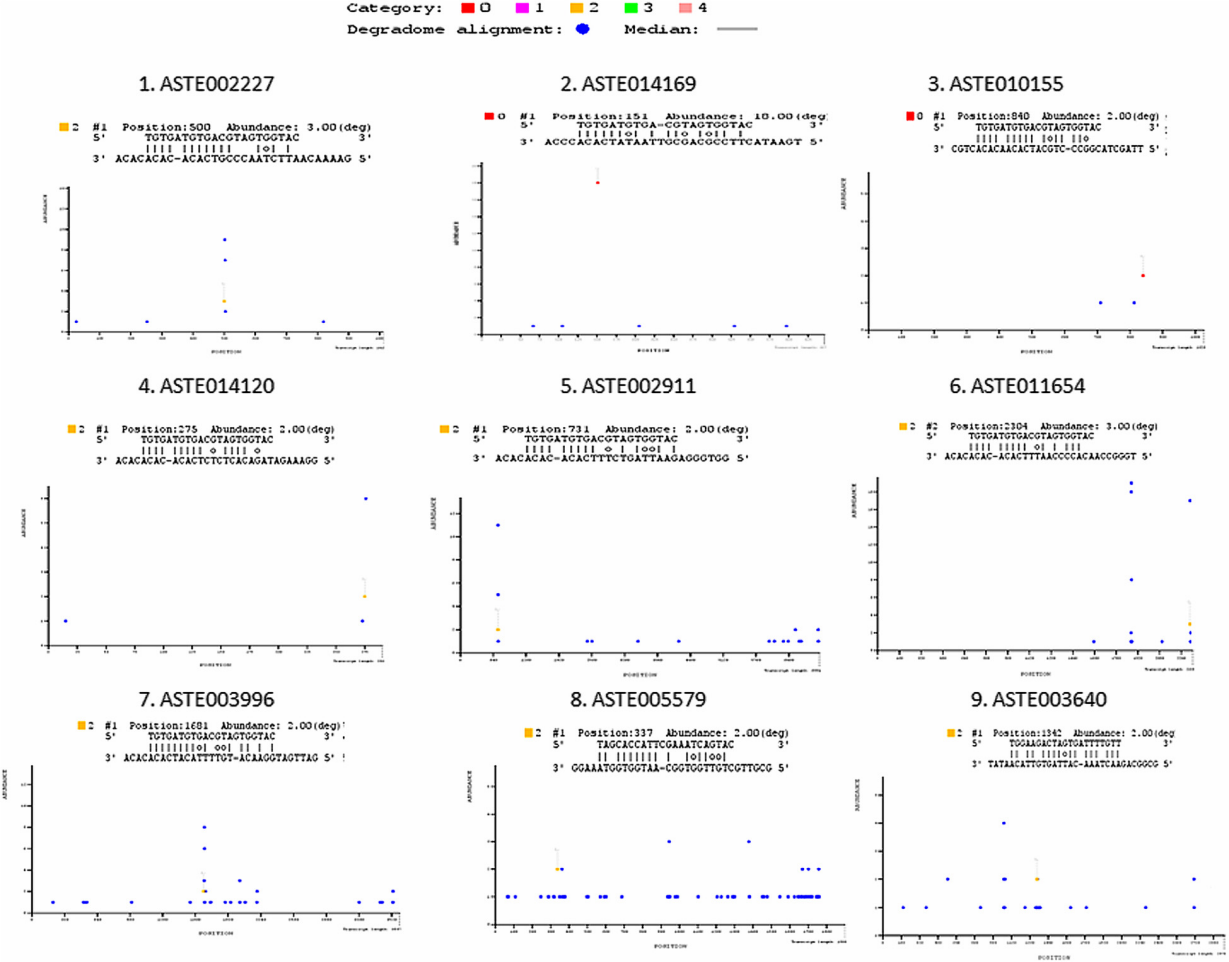
**miRNA targets identification by Degradome sequencing.** T-plots of miRNA targets classified under category 2, 1 and 0. X-axis represents transcript length whereas Y-axis represents number of degradome read that were mapped on to specific position on the target sequence. Blue dot depicts position on the transcript where degraded read was mapped. Coloured dots represent the position on the transcript where degraded fragment (s) and miRNA seed sequence mapped on the transcript.

Out of 29 total targets cleaved by all six miRNAs, 19 were classified under category 4, one under category 3, seven under category 2 and two targets were classified under category 0. None of these targets were classified under category 1. T-plots of targets falling under category 2, 1 and 0 are shown in Figure 5. GO terms of these targets are provided in Table 5.

Out of 29 targets identified, GO term name were identified for 14 mRNAs (Table 5). GO terms of these mRNAs were enriched in pathways related to protein binding and proteolysis. These targets might be important for insect reproduction as protein deposition in the ovary results in maturation of the developing egg. Another GO term significantly represented in this cluster of targets was related to nucleic acid binding. Nucleic acid binding might be responsible proper transcription and translation of genes required for maternal to zygotic transition in developing eggs.

## Discussion

*An.stephensi* is an important vector of malaria parasite and is distributed throughout the Middle East and South Asia region. Various strategies were planned to control malaria spread, few of which were directed towards controlling the expansion of vector population. Such strategies require detailed understanding of mosquito biology pertaining to its developmental stages. Mosquito being a holometabolous insect goes through four stages of development. All four stages show characteristic morphological and physiological differences, resulting from differential gene expression at each stage of development. To understand molecular mechanisms mediating such differences, we studied the stage specific differences in miRNA expression which are known regulators of gene expression.

In this study, we employed deep sequencing technology to identify miRNAs expressed during mosquito development. Many of the 111 known miRNAs were same as those identified in our previous study [9]. Nevertheless, few more known miRNAs were added to the list that were not identified earlier in blood fed and parasitized mosquito [9]. Three microRNAs (miR-8, miR-bantam and miR-281) were abundantly expressed across all developmental stages of development indicating house-keeping function of these miRNAs. In *Drosophila*, miR-bantam functions in maintaining circadian rhythm and controls cell proliferation [26,27]. MicroRNA-8 regulates PI3K activity in larval fat boy and controls apoptosis/neurodegeneration in insects [28,29]. MicroRNA-281 was shown to be involved in regulating dengue virus replication in *Aedes albopictus* [30]. Efforts were also taken to identify novel mosquito-specific miRNAs in *An. stephensi.* Seven pairs of small RNA reads (−5p and -3p) fulfilled set criteria’s to be classified as mature miRNAs [31]. Expression values of each miRNA in all six libraries were normalised and TPM vales were compared to study miRNAs regulation across mosquito developmental stages. Understanding the differences between male and female mosquito is the most significant as these differences make female mosquitoes fit for reproduction and parasite transmission. Therefore, we identified miRNAs that were differentially regulated between genders during larval, pupal and adult stages of development. Few miRNAs were differentially expressed between genders during larval and pupal stages as these stages are early stages of development and differences between genders are not very apparent. On the other hand, six miRNAs were found differentially expressed between male and female adult mosquito. Out of these six miRNAs, miR-989 was of significant importance as it was reported to get abundantly expressed in ovary of female mosquito [16]. Its expression levels were up-regulated in female mosquito post blood feeding indicating towards its role in mosquito reproduction [9]. Two microRNAs, miR-989 and miR-7 also showed gender difference expression in *Anopheles anthropophagus* [14]*.*

This is the first extensive study wherein efforts were taken to understand miRNA regulation during complete metamorphosis in male and female mosquito. MicroRNA differences were studied during larva to pupa transition and from pupa to adult metamorphosis in both male and female mosquito. Larva undergoes molting or ecdysis resulting in its growth and development from first to fourth instar larva. Histolysis and phagocytosis of larval tissues results in larval transition to pupa stage. Many miRNAs were found differentially expressed between these two stages. Few miRNAs were common in male and female metamorphosis from larva to pupa stage. While, few miRNAs regulated differently during male and female metamorphosis were identified. The role of few of these regulated miRNAs has been shown in *Drosophila*. Let-7 was required for neuromuscular remodeling and temporal organization during metamorphosis [32,33]. Another regulated miRNA, miR-7 was involved in notch signalling and photoreceptor differentiation in *Drosophila* eye [34,35]. Metamorphosis of pupa to adult mosquito involves tissue lysis and re-organization resulting in formation of adult organs. This process involves complex interplay between molecular factors as indicated by significant number of miRNAs found regulated during pupa to adult mosquito metamorphosis. MicroRNA-989 was identified up-regulated in female mosquito whereas it was down-regulated in male mosquito during the transition. It was found specifically up-regulated in female mosquito when compared to other stages of mosquito development. Such observation highlights its function in physiological activities related specifically with adult female mosquito. Regulated miRNAs such as miR-34 and miR-9a were involved in various processes involved in insect development [36-39]*.*

Development of complex organisms requires controlled balance between mRNA degradation and translation. This fine balance is maintained by many factors including miRNAs. To understand the role of regulated miRNAs in mosquito development, it is necessary to identify their mRNA targets. As miRNAs binds to 3’UTR of targets sequence and regulate their expression, efforts were taken to identify miRNA seed binding sites on 3’UTR sequence of *An. stephensi* genes. Many targets of each miRNAs were identified using RNA hybrid. Targets of miRNA regulated at specific stage were analysed together and enriched annotation clusters playing significant role at specific stage of development were identified. Pathways involved in metabolic processes, phagocytosis and membrane organization were found enriched during larval to pupal transition. These pathways might be important for histolysis and phagocytosis of larval tissue which results in its transition to pupal stage. Metamorphosis from pupal to adult stage involves pathways such as vesicle mediated transport, phagocytosis, protein and polysaccharide biosynthetic processes. Genes functional in translation and enzymatic activity were also found enriched during mosquito metamorphosis. These pathways clearly are required for pupa tissue re-organization and re-formation of adult mosquito organs.

In our study, we identified miRNAs that were differentially expressed in male and female adult mosquitoes. Several studies have emphasised the role of some of these regulated miRNAs in female mosquitoes especially upon blood feeding [9,16]. In an attempt to identify targets of miRNAs regulated in our study that could have a possible role in female mosquitoes, ovary tissues were analysed upon blood feeding to identify targets, which could further be studied using miRNA specific antagomirs. This will be helpful to elucidate miRNA biological functions in an organism [12,25]. In this study, we inhibited miR-989 expression by injecting antagomir in mosquitoes. MicroRNA-989 up-regulates in female mosquito compared to male mosquito. Its expression further increases in ovary tissue of mosquito post blood feeding highlighting towards its significant role in mosquito reproduction [16]. Inhibition of miRNA expression via antagomir was combined with degradome sequencing to identify and validate miR-989 targets in ovary of blood fed female mosquito. Cleaved miRNA targets have been identified in plant kingdom using degradome sequencing [40,41]. This is the first report to have used degradome sequencing for miRNA targets identification in insects. Degradome sequencing has been employed mainly for identification of miRNA targets in plants as these miRNAs bind to their target sequence with perfect complementarity resulting in their cleavage [40,41]. Therefore, we identified very few cleaved targets in mosquitoes as miRNAs in animal kingdom generally regulate gene expression by mRNA translational repression. Targets were identified in ovary tissue of mosquito by degradome sequencing for miRNAs differentially expressed between both genders. Differentially expressed miRNAs resulted in regulation of twenty nine targets by mediating their cleavage in ovary of female mosquito. Many targets have GO term related to protein binding and proteolysis which might be important for egg development that requires massive protein deposition in developing embryo. Also targets with GO term related to metabolic processes, nucleic acid binding and signal transduction were identified. Functional characterizations of these targets are underway to understand their role in mosquito developmental processes. Deciphering function of many cleaved targets identified in ovary tissue would provide us with a better idea about their role in mosquito reproduction.

## Conclusions

In conclusion, this study provides comprehensive account of miRNA regulated across different stages of mosquito development. Efforts were taken to understand targets of these miRNAs which can provide better understanding of their biological function in mosquito development. Identification of miRNA targets in ovary of blood fed female mosquito could provide insights in mosquito reproductive process and has implications for effective control of mosquito population required for reducing spread of mosquito-borne infectious diseases.

## Additional files

Additional file 1:
**Predicted stem-loop structures of Novel miRNAs.** Precursor structures of mature miRNAs were folded using RNA fold. Sequences forming stem-loop structures with folding energy < −20 kcal/mol and with mature miRNAs sequences lying on the stem region were selected as Novel miRNAs. Additional file 2:
**miRNAs regulated between two genders across different stages of their development.** (A) Heat map of miRNAs differentially expressed between larva male (LM) and larva female (LF), pupa male (PM) and pupa female (PF), adult male (AM) and adult female (AF) mosquito. Colour gradation from light green to dark red represents relative increase in miRNA expression. (B) Column graph showing fold change in miRNAs expression between larva male (LM) and larva female (LF) mosquito. (C) Column graph showing fold change in miRNAs expression between pupa male (PM) and pupa female (PF) mosquito. (D) Column graph showing fold change in miRNAs expression between adult male (AM) and adult female (AF) mosquito. Additional file 3:
**miRNAs regulated across different stages of male mosquito development.** (A) Heat map of miRNAs differentially expressed between larva male (LM), pupa male (PM) and adult male (AM) mosquito. Colour gradation from light green to dark red represents relative increase in miRNA expression. (B) Column graph showing fold change in miRNAs expression between larva male (LM) and pupa male (PM) mosquito. (C) Column graph showing fold change in miRNAs expression between pupa male (PM) and adult male (AM) mosquito. Additional file 4:
**miRNAs regulated across different stages of female mosquito development.** (A) Heat map of miRNAs differentially expressed between larva female (LF), pupa female (PF) and adult female (AF) mosquito. Colour gradation from light green to dark red represents relative increase in miRNA expression. (B) Column graph showing fold change in miRNAs expression between larva female (LF) and pupa female (PF) mosquito. (C) Column graph showing fold change in miRNAs expression between pupa female (PF) and adult female (AF) mosquito. Additional file 5:
**List of miRNA targets identified by RNAhybrid on 3’UTR of** ***An.stephensi*** **genes.**

## Abbreviations

miRNA: microRNA
Pre-miRNA: Precursor microRNA
AF: Adult female
AM: Adult male
LM: Larva male
LF: Larva female
PM: Pupa male
PF: Pupa female
piRNA: Piwi-interacting RNA
nt: Nucleotide
UTR: Untranslated region
NGS: Next generation sequencing
TPM: Tags per million
LNA: Locked nucleic acid
bp: Base pair
mRNA: Messenger RNA
rRNA: Ribosomal RNA
tRNA: Transfer RNA
snoRNA: Small nucleolar RNA
RT-PCR: Real time polymerase chain reaction
EDC: l-ethyl-3-(3-dimethylaminopropyl) carbodiimide
SSC: Saline sodium citrate
