# Supplementary figures and images for: Dynamic expression of miRNAs across immature and adult stages of the malaria mosquito Anopheles stephensi
Source: Parasit Vectors. 2015 Mar 25;8:179. doi: 10.1186/s13071-015-0772-y (PMC4418096; doi:10.1186/s13071-015-0772-y)

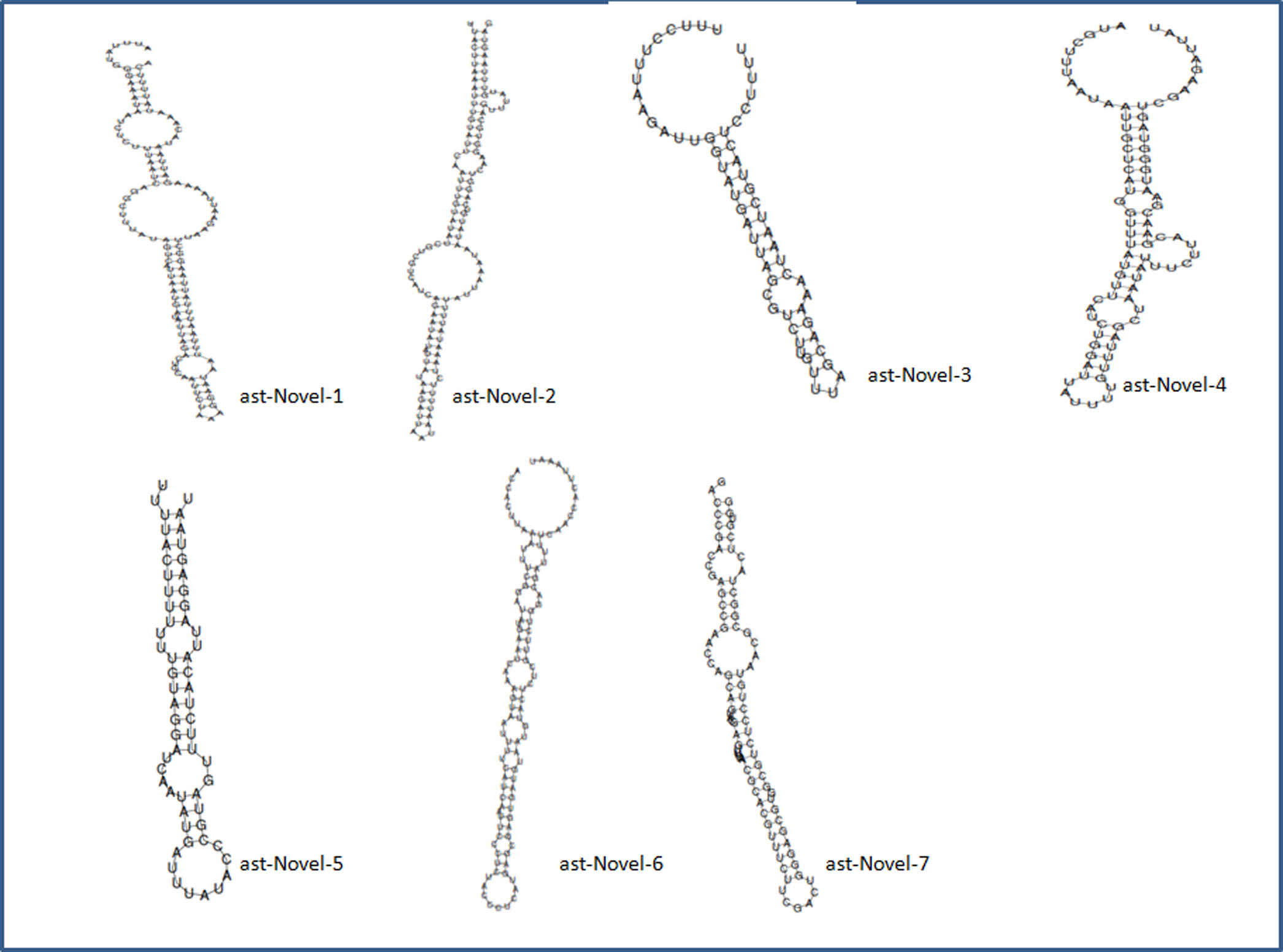

Supplement: Additional file 1: — Predicted stem-loop structures of Novel miRNAs. Precursor structures of mature miRNAs were folded using RNA fold. Sequences forming stem-loop structures with folding energy < −20 kcal/mol and with mature miRNAs sequences lying on the stem region were selected as Novel miRNAs. [file 13071_2015_772_MOESM1_ESM.jpg]

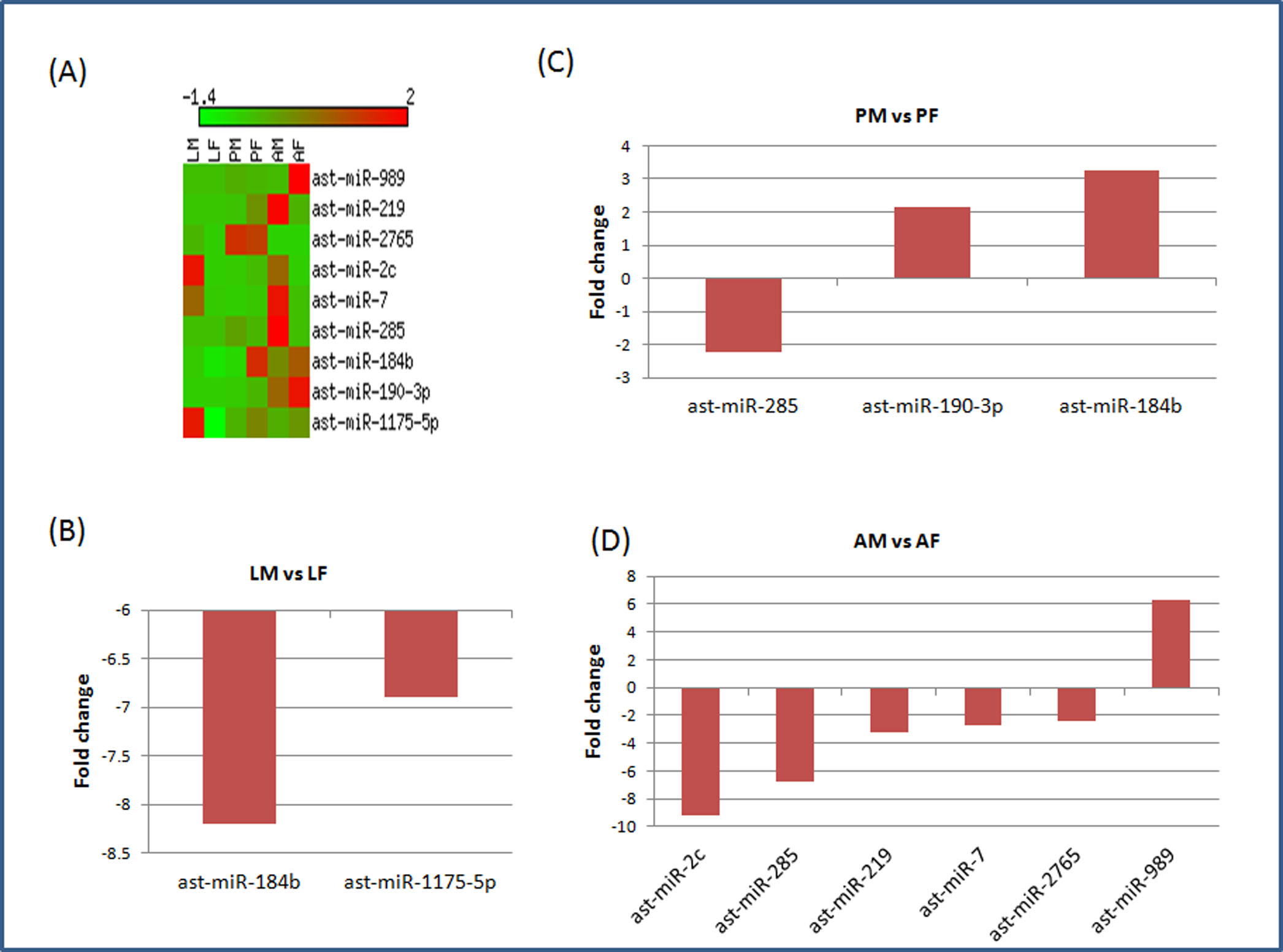

Supplement: Additional file 2: — miRNAs regulated between two genders across different stages of their development. (A) Heat map of miRNAs differentially expressed between larva male (LM) and larva female (LF), pupa male (PM) and pupa female (PF), adult male (AM) and adult female (AF) mosquito. Colour gradation from light green to dark red represents relative increase in miRNA expression. (B) Column graph showing fold change in miRNAs expression between larva male (LM) and larva female (LF) mosquito. (C) Column graph showing fold change in miRNAs expression between pupa male (PM) and pupa female (PF) mosquito. (D) Column graph showing fold change in miRNAs expression between adult male (AM) and adult female (AF) mosquito. [file 13071_2015_772_MOESM2_ESM.jpg]

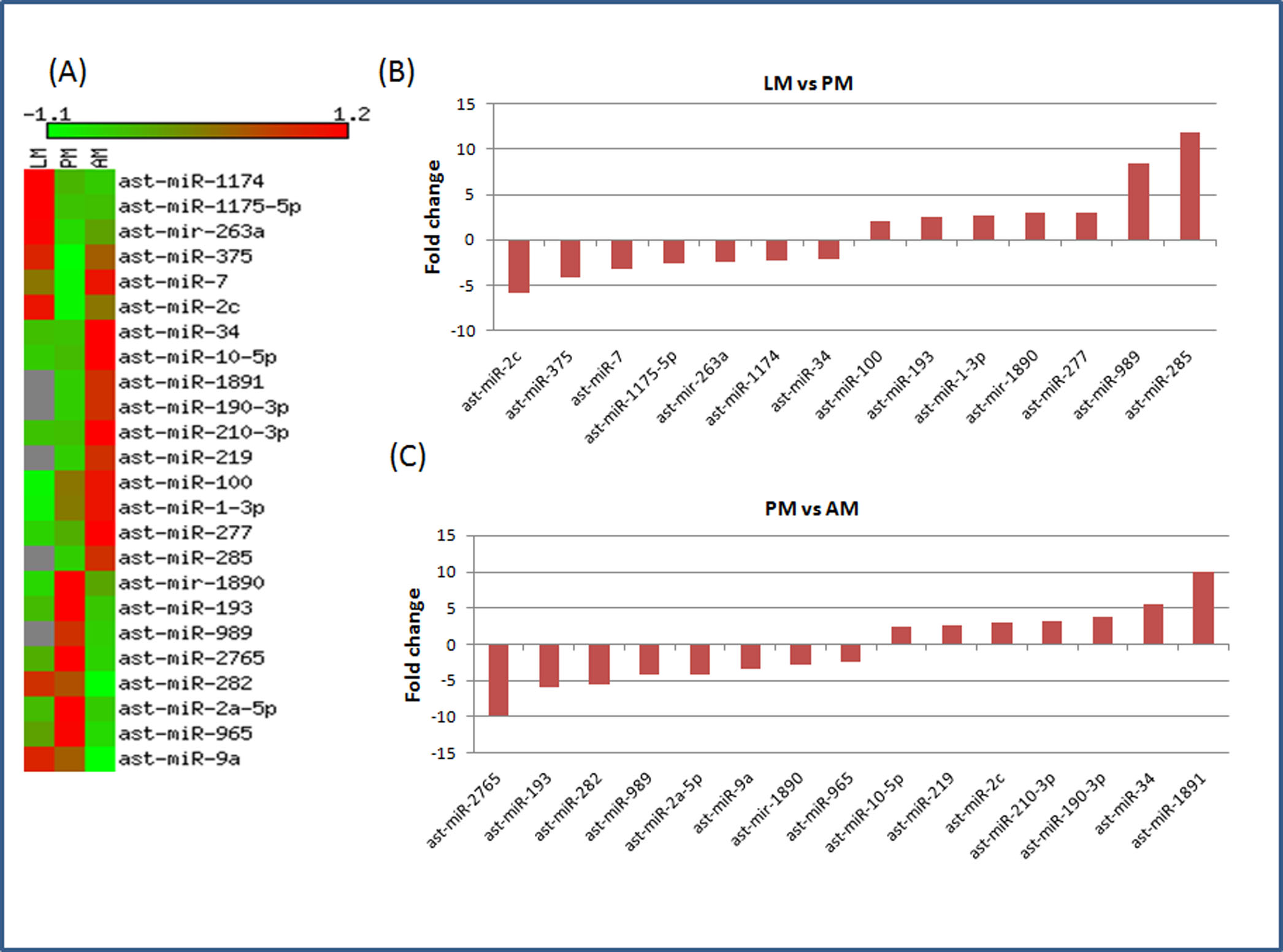

Supplement: Additional file 3: — miRNAs regulated across different stages of male mosquito development. (A) Heat map of miRNAs differentially expressed between larva male (LM), pupa male (PM) and adult male (AM) mosquito. Colour gradation from light green to dark red represents relative increase in miRNA expression. (B) Column graph showing fold change in miRNAs expression between larva male (LM) and pupa male (PM) mosquito. (C) Column graph showing fold change in miRNAs expression between pupa male (PM) and adult male (AM) mosquito. [file 13071_2015_772_MOESM3_ESM.jpg]

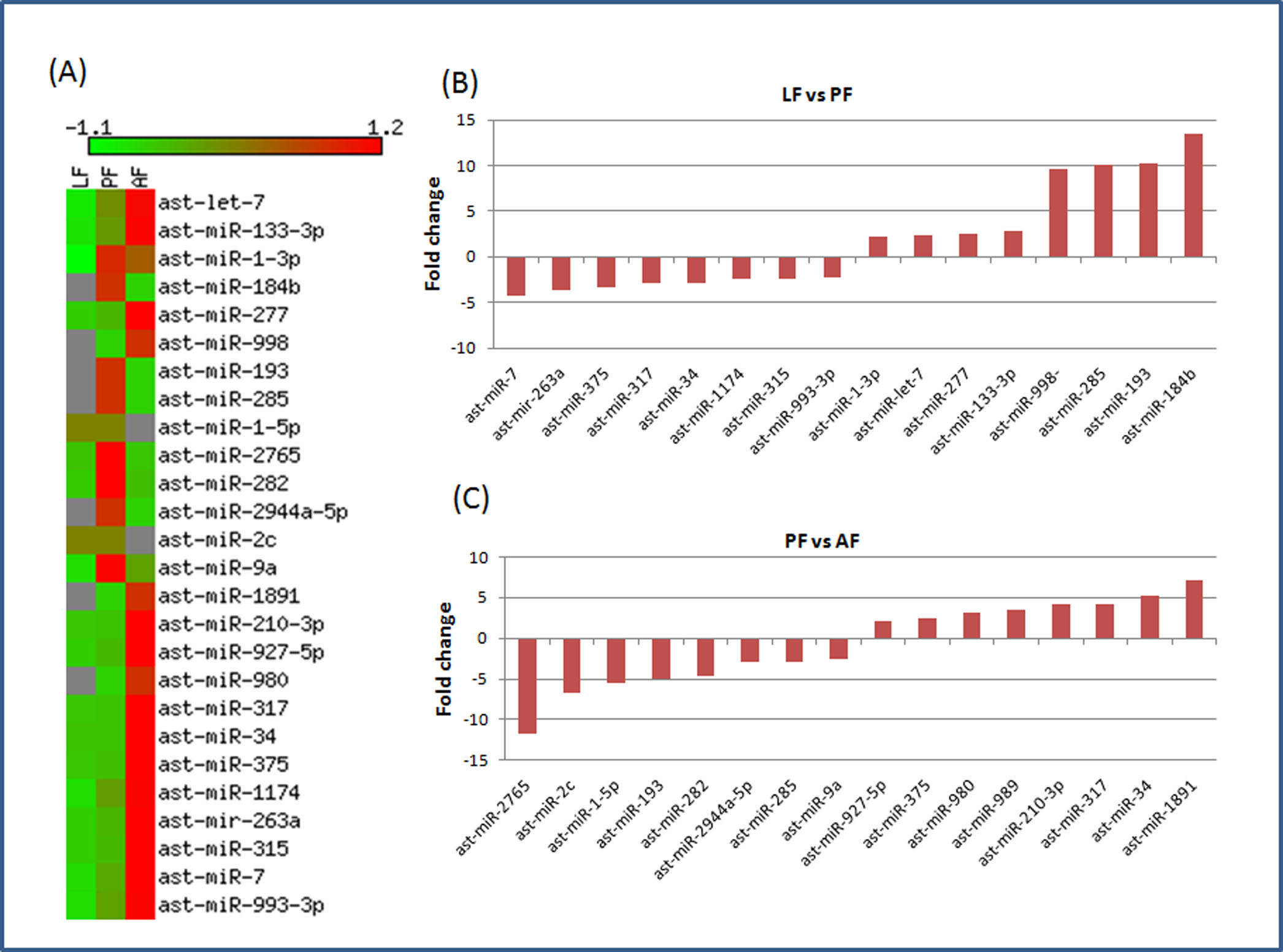

Supplement: Additional file 4: — miRNAs regulated across different stages of female mosquito development. (A) Heat map of miRNAs differentially expressed between larva female (LF), pupa female (PF) and adult female (AF) mosquito. Colour gradation from light green to dark red represents relative increase in miRNA expression. (B) Column graph showing fold change in miRNAs expression between larva female (LF) and pupa female (PF) mosquito. (C) Column graph showing fold change in miRNAs expression between pupa female (PF) and adult female (AF) mosquito. [file 13071_2015_772_MOESM4_ESM.jpg]
